# IL-37 Ameliorating Allergic Inflammation in Atopic Dermatitis Through Regulating Microbiota and AMPK-mTOR Signaling Pathway-Modulated Autophagy Mechanism

**DOI:** 10.3389/fimmu.2020.00752

**Published:** 2020-04-28

**Authors:** Tianheng Hou, Xiaoyu Sun, Jing Zhu, Kam-Lun Hon, Peiyong Jiang, Ida Miu-Ting Chu, Miranda Sin-Man Tsang, Christopher Wai-Kei Lam, Huasong Zeng, Chun-Kwok Wong

**Affiliations:** ^1^Department of Chemical Pathology, The Chinese University of Hong Kong, Hong Kong, China; ^2^Department of Paediatrics, The Chinese University of Hong Kong, Hong Kong, China; ^3^State Key Laboratory of Research on Bioactivities and Clinical Applications of Medicinal Plants, Institute of Chinese Medicine, The Chinese University of Hong Kong, Hong Kong, China; ^4^State Key Laboratory of Quality Research in Chinese Medicines, Faculty of Medicine, Macau University of Science and Technology, Macau, China; ^5^Department of Allergy, Immunology and Rheumatology, Guangzhou Women and Children’s Medical Center, Guangzhou Medical University, Guangzhou, China

**Keywords:** allergic inflammation, atopic dermatitis, autophagy, eosinophils, IL-37, microbiota

## Abstract

Interaction between eosinophils and dermal fibroblasts is essential for provoking allergic inflammation in atopic dermatitis (AD). *In vitro* co-culture of human eosinophils and dermal fibroblasts upon AD-related IL-31 and IL-33 stimulation, and *in vivo* MC903-induced AD murine model were employed to investigate the anti-inflammatory mechanism of IL-1 family cytokine IL-37 in AD. Results showed that IL-37b could inhibit the *in vitro* induction of AD-related pro-inflammatory cytokines IL-6 and TNF-α, and chemokines CXCL8, CCL2 and CCL5, increase autophagosome biogenesis-related LC3B, and decrease autophagy-associated ubiquitinated protein p62 by regulating intracellular AMP-activated protein kinase (AMPK) and mammalian target of rapamycin (mTOR) signaling pathway. In CRISPR/Cas9 human IL-37b knock-in mice, IL-37b could significantly alleviate MC903-stimulated ear tissue swelling, itching sensation and the level of circulating cytokine IL-6 and ear *in situ* expression of AD-related TNF-α, CCL5 and transforming growth factor-β. Moreover, IL-37b could significantly upregulate Foxp3+ regulatory T cells (Treg) in spleen and ear together with significantly increased serum Treg cytokine IL-10, and decrease eosinophil infiltration in ear lesion. IL-37b knock-in mice showed a distinct intestinal microbiota metabolic pattern upon MC903 stimulation. Furthermore, IL-37b restored the disordered gut microbiota diversity, through regulating the *in vivo* autophagy mechanism mediated by intestinal metabolite 3-methyladenine, adenosine monophosphate, 2-hydroxyglutarate, purine and melatonin. In summary, IL-37b could significantly ameliorate eosinophils-mediated allergic inflammation via the regulation of autophagy mechanism, intestinal bacterial diversity and their metabolites in AD. Results therefore suggest that IL-37 is a potential anti-inflammatory cytokine for AD treatment.

## Introduction

Atopic dermatitis (AD) is a chronic relapsing inflammatory skin disease, characterized by skin lesions, pruritic excoriations and susceptibility to cutaneous infections (1). There is no definitive cure for AD and the current treatment with immunosuppressive corticosteroid is associated with undesirable side effects. Eosinophils (EOS), as the principal effector cells of allergic inflammation including AD (2), their accumulation and infiltration in tissues are mediated by the specific eosinophil chemokine eotaxin, vascular cell adhesion molecule-1, intercellular adhesion molecule-1 and E-selectin (2, 3).

IL-1 family cytokine IL-37/IL-1F7 can downregulate systemic and local inflammation and Th2 cytokines, by suppressing the production of pro-inflammatory mediators and cytokines in innate and adaptive immunity (4). Clinical studies have revealed that IL-37 levels in serum and skin tissue were significantly higher in AD patients compared with controls, thereby implicating the induction of IL-37 by skin barrier disruption (5). IL-37 can be expressed by regulatory T cells (Treg) to enhance the expression of IL-10, Foxp3, and cytotoxic T-lymphocyte associated antigen-4 to promote the immunosuppressive activity of human Treg cells (6). Receptor IL-18Rα and IL-1R8 can mediate the multifaceted anti-inflammatory activity of IL-37, via the regulation of cellular adhesion and migration, and intracellular signal transduction mechanism (4, 7). Among the five different IL-37 splice variants, IL-37b is the most effective and best characterized variant (8). This study was performed to investigate the anti-inflammatory mechanisms of IL-37 in AD by using recombinant human IL-37b and CRISPR/Cas9 human IL-37b knock-in mice.

IL-37 has been reported to alleviate inflammation by regulating intracellular AMP-activated protein kinase (AMPK)-mammalian target of rapamycin (mTOR) signaling mechanism, which is the key regulator of autophagy (9). Autophagy has been extensively reported to play a vital role in regulating inflammation of the immune system (10). It is the key player in resistance to bacterial, viral and protozoan infections (11) because the autophagy action mechanism can eliminate numerous unbeneficial microbes such as Streptococcus pyogenes (12). Gut microbiota (GM) can play a critical role in shaping the development of the host immune system in early life (13). Early exposure to GM can foster the shift of Th1/Th2 balance to Th1 phenotype. In contrast, abnormal intestinal colonization in atopic diseases, especially in the development of the mucosal immune system, can shift the Th1/Th2 balance to Th2 response (14). Th2 cell-derived cytokines such as IL-4, IL-5, and IL-13, induce the class switch of immunoglobulins to IgE, thereby mediating the allergic response. In addition, metabolites secreted by microbiota, e.g., short chain fatty acids such as butyrate, acetate and propionate, have been shown to control the differentiation and function of mucosal Tregs cells (15).

In the present study, the mechanism of detailed immunoregulatory role of IL-37b in Th2-related eosinophils-mediated interaction with dermal fibroblasts in AD was explored using *in vitro* and *in vivo* experiments. Further non-targeted metabolomic analysis and GM profile of CRISPR/Cas9 human IL-37b knock-in and wild type mice with AD were employed to elucidate the anti-inflammatory mechanism of IL-37 in AD.

## Materials and Methods

### Mice

Inbred CRISPR/Cas9 human IL-37b knock-in mice (8 weeks old; C57BL/6 background) were purchased from Cyagen Biosciences (Guangzhou) Inc, China.

### AD Mouse Model

The AD mouse model was established using MC903 stimulation for 16 days. A total of 2 nmol of MC903 (Sigma-Aldrich Corp, St. Louis, MO, United States) was topically applied in 5 μL of ethanol to one ear of a mouse every other day for 16 days. Ear thickness was measured with a dial thickness gauge (Model G, Peacock, Ozakimfg Co, Ltd, Tokyo, Japan) and snatching time within 5 min were recorded to assess itching severity every other day for 17 days, followed by sacrificing the mice for post mortem analysis of AD skin lesions. Quick and continuous multiple scratching within a very short period was considered as one-time scratching. All animal experiments were approved by the Animal Experimentation Ethics Committee of the Chinese University of Hong Kong, Hong Kong.

### Histological Examination and Immunohistofluorescence Study

Sections (5 mm) were stained with H&E to assess the general morphology. Paraffin sections were immunostained with lineage-specific antibodies to identify eosinophils and dermal fibroblasts by immunohistofluorescence. Samples were incubated with rat anti-mouse major basic protein (MBP) antibody (specific for eosinophils, a gift from James J. Lee, Ph.D., Mayo Clinic, Scottsdale, AZ, United States), rabbit anti-Vimentin antibody (specific for dermal fibroblasts, Cell Signaling Technology, Beverly, MA, United States), AMPK alpha-1 monoclonal antibody, mTOR monoclonal antibody, and IL-37 polyclonal antibody (Thermo Fisher Scientific, Rockford, IL, United States), and LC3B antibody (Sigma-Aldrich, St. Louis, MO, United States). Cy3-conjugated goat anti-rat immunoglobulin G (IgG) antibody (Beyotime Co., Shanghai, China) and Alexa Fluor 488-conjugated goat anti-rabbit and goat anti-mouse IgG antibody (ABclonal, MA, United States) were used as secondary antibodies. All images were acquired with a Leica DM6000B microscope (Leica Microsystems GmbH, Wetzlar, Germany) and processed using the Leica Application Suite software (Leica Microsystems GmbH).

### Preparation of Single-Cell Suspensions and Flow Cytometric Analysis

Single-cell suspensions were prepared from spleens of wild type and IL-37b Tg mice. Spleen was mechanically disrupted and homogenate collected by using a 70 μm cell strainer (Corning Inc., New York, NY, United States). Single splenic cells were washed with wash buffer (1 x PBS supplemented with 2 mM EDTA and 2% FBS), and analyzed using a FACSVia flow cytometer (BD Biosciences, San Jose, CA, United States) with a mouse Treg cell staining kit (Invitrogen).

### RNA Extraction and Quantitative RT-PCR

Total RNA of mouse ear tissue (10 mg) was extracted using RNA extraction kit (Qiagen Corp., Germantown, MD, United States). cDNA was synthesized using SuperScript II Reverse Transcriptase (Invitrogen). Quantitative real-time RT-PCR analysis of the cDNA was performed using a StepOnePlus Real-Time PCR System (Thermo Fisher Scientific, Rockford, IL, United States) with SYBR Green Master Mix (Bio-Rad). The relative mRNA expression of each gene was determined using ddCt calculation method with GAPDH as internal housekeeping gene.

Primer sequences used were: IL-4: Forward 5′-ACAGGA GAAGGGACGCCAT-3′, Reverse 5′-ACCTTGGAAGCCCTAC AGA-3′; CCL2: Forward 5′-GCATCTGCCCTAAGGTCTTCA-3′, Reverse 5′-GTGGAAAAGGTAGTGGATGCATT-3′; Foxp3: Forward 5′-CCCAGGAAAGACAGCAACCTT-3′, Reverse 5′-T TCTCACAACCAGGCCACTTG-3′; TNF-α: Forward 5′-CACA GAAAGCATGATCCGCGACGT-3′, Reverse 5′-CGGCAGA GAGGAGGTTGACTTTCT-3′; TGF-β: Forward 5′-CACAGAA AGCATGATCCGCGACGT-3′, Reverse 5′-CGGCAGAGAGGA GGTTGACTTTCT-3′; CCR3: Forward 5′-AAGCTTTGAGAC CACACCCTATG- 3′, Reverse 5′-GACCCCAGCTCTTTGATT CTGA-3′; CCL5: Forward 5′-CCCTCACCATCATCCTCACT-3′, Reverse 5′-TCCTTCGAGTGACAAACACG-3′; mGAPDH: Forward 5′-TGGTGAAGCAGGCATCTGAG-3′, Reverse 5′-TG TTGAAGTCGCAGGAGACAAC-3′.

### Purification of Eosinophils

Human eosinophils were purified from fresh human buffy coats (Hong Kong Red Cross Blood Transfusion Service) using anti-CD16 magnetic beads and LS+ column within a magnetic field (Miltenyi Biotec). Purity of eosinophils (at least 96%) was assessed by Hemacolor rapid blood smear stain (E Merck Diagnostica, Darmstadt, Germany). The above protocol using human eosinophils was approved by Clinical Research Ethics Committee, The Chinese University of Hong Kong-New Territories East Cluster Hospitals according to the Declaration of Helsinki.

### Co-culture of Human Eosinophils and Dermal Fibroblasts

Primary human dermal fibroblasts (Life Technologies, Carlsbad, CA, United States) were grown in Medium 106 supplemented with low serum growth supplement (Gibco Invitrogen Corp., Carlsbad, CA, United States). Eosinophils (3 × 10^5^ cells) and confluent fibroblasts (1 × 10^5^ cells) were co-cultured in RPMI 1640 medium containing 10% FBS with or without IL-37b (100 ng/mL) pre-treatment for 10 mins, followed by IL-31 and IL-33 (100 ng/mL) stimulation for 24 h prior to the determination of the expression of cytokines/chemokines and autophagy related proteins.

### Autophagy Analysis

Co-culture of human eosinophils and dermal fibroblasts was treated with IL-31 and IL-33 (100 ng/mL), rapamycin (10 ng/mL), IL-37b (100 ng/mL), or chloroquine (20 μM) for 1, 2, 4, and 16 h. Cells were stained and autophagic vacuoles were detected at indicated time by using Autophagy Detection Kit (Abcam, Cambridge, United Kingdom). Fluorescent autophagic vacuoles were analyzed by a Leica DM6000B fluorescence microscope (Leica Microsystems GmbH, Wetzlar, Germany).

### Quantification of Cytokines and Chemokines

Concentrations of human inflammatory cytokines and chemokines including TNF-α, IL-6, CCL2, CCL5, and CXCL8 in culture supernatant were measured using the cytometric bead array kit (BD Biosciences, San Jose, CA, United States) on a BD FACSVia flow cytometer. Murine interferon IL-5, IL-13, IL-6, and IL-10 in serum were quantified with mouse cytokine Milliplex MAP assay kit (Millipore Corporation, Billerica MA, United States) using the Bio-Plex 200 system (Bio-Rad Laboratories, Hercules, CA, United States).

### Western Blot

Cells from the co-culture and homogenized mouse ear tissue were lysed with RIPA lysis buffer (Thermo Fisher Scientific). Protein content from cell lysis and mouse ear tissue was determined with protease inhibitor and phosphatase inhibitor (Sigma-Aldrich) and Pierce BCA protein assay (Thermo Fisher Scientific). AMPK alpha-1 monoclonal antibody, mTOR monoclonal antibody and IL-37 polyclonal antibody were obtained from Thermo Fisher Scientific. SQSTM1/P62, β-actin rabbit mAb, HRP-conjugated anti-rabbit IgG and HRP-conjugated anti-mouse IgG were obtained from Cell Signaling Technology. Dorsomorphin (Compound C/CC AMPK inhibitor) and LC3B were obtained from Sigma-Aldrich Corp. Secondary antibodies were used at 1:2000 and other primary antibodies were diluted at 1:1000.

### Metabolomic Studies

Stools (25 mg) obtained from mice at day 1, 15, and 17 were individually grounded with liquid nitrogen and the homogenate was resuspended with prechilled 80% methanol and 0.1% formic acid. The collected supernatant was diluted to a final concentration containing 60% methanol. LC-MS/MS analysis was performed using a Vanquish UPLC system (Thermo Fisher Scientific) coupled with an Orbitrap Q Exactive HF-X mass spectrometer (Thermo Fisher Scientific). Samples were injected onto a Hyperil Gold column (100 × 2.1 mm, 1.9 μm) using a 16-min linear gradient at a flow rate of 0.2 mL/min. The eluents for positive polarity mode were eluent A (0.1% FA in Water) and eluent B (Methanol). The eluents for negative polarity mode were eluent A (5 mM ammonium acetate, pH 9.0) and eluent B (methanol). The solvent gradient was set as follows: 2% B, 1.5 min; 2–100% B, 12.0 min; 100% B, 14.0 min; 100-2% B, 14.1 min; 2% B, 16 min. Q Exactive HF-X mass spectrometer was operated in positive/negative polarity mode with spray voltage of 3.2 kV, capillary temperature of 320°C, sheath gas flow rate of 35 arb and aux gas flow rate of 10 arb. Raw data files generated by UPLC-MS/MS were processed to perform peak alignment, peak picking and quantitation for each metabolite by using Compound Discoverer 3.0 (CD 3.0, Thermo Fisher Scientific). Peak intensities were normalized to predict the molecular formulas and metabolites based on additive ions, molecular ion peaks and fragment ions by matching with mzCloud^1^ and ChemSpider^2^ database.

### 16S rRNA Sequence

Total genome DNA from stool samples was extracted using CTAB/SDS method. DNA was diluted to 1 ng/μL using sterile water. 16S rRNA genes of distinct regions (16S V3-V4) were amplified using specific primer (341F 5′-CCTAYGGGRBGCASCAG-3′, 806R 5′-GGACTACNNGGGTA TCTAAT-3′) with the barcode. All PCR reactions were carried out with Phusion^®^ High-Fidelity PCR Master Mix (New England Biolabs). PCR products were purified with GeneJETTM Gel Extraction Kit (Thermo Fisher Scientific). Sequencing libraries were generated using Ion Plus Fragment Library Kit (Thermo Fisher Scientific) following the manufacturer’s recommendations, and quality control was conducted with the Qubit@ 2.0 Fluorometer (Thermo Fisher Scientific). 400/600bp single-end reads were generated on an Ion S5TM XL platform. 16S rRNA amplification and sequencing service were provided by Novogene Co., Ltd., Beijing, China.

Alpha diversity and beta diversity on both weighted and unweighted unifrac were calculated by QIIME software (Version1.7.0) and displayed with R software (Version 2.15.3) using Principal Component Analysis (PCA), Non-Metric Multi-Dimensional Scaling (NMDS) and Unweighted Pair-group Method with Arithmetic Means (UPGMA) analysis. Partial least-square discriminant analysis (PLS-DA) was performed to discriminate the microbial community profiles among groups. The metagenomic sequencing data (16S rRNA) has been deposited in European Nucleotide Archive (ENA) with accession number PRJEB36050.

### Statistical Analysis

Data were presented as mean ± SEM, and analyzed by one-way analysis of variance (ANOVA) followed by Dunnett’s test for multiple inter-group comparisons using GraphPad PRISM software version 6.01. *P* < 0.05 was considered statistically significant.

## Results

### Anti-inflammatory Effect of IL-37b on the Release of Cytokines/Chemokines in Eosinophil-Dermal Fibroblast Co-culture Upon IL-31 and IL-33 Stimulation

Since IL-18Rα and IL-1R8 are receptors for mediating the anti-inflammatory activities of IL-37b (7), both IL-18Rα and IL-1R8 were detected on the cell surface of human dermal fibroblast (HDF) cells by flow cytometry (Supplementary Figure 1). Our previous studies have shown that IL-18Rα and IL-1R8 were expressed on the surface of eosinophils (16). MTT cytotoxicity results demonstrated that IL-37b (10 – 1,000 ng/ml) did not exhibit any remarkable effect (*P* > 0.05) on both cell proliferation and viability (Supplementary Figure 2).

IL-37b treatment suppressed the *in vitro* IL-31 and IL-33-induced TNF-α, IL-6, CXCL8, CCL2 and CCL5 in the co-culture of EOS and HDF (Supplementary Figures 3, 4). IL-37b significantly downregulated CCL2 and CXCL8 production upon IL-31 and IL-33 stimulation in HDF-alone culture supernatant (*P* < 0.05, Supplementary Figures 3C,D). Co-culture system produced significantly higher release of inflammatory cytokines and chemokines upon IL-31 and IL-33 stimulation compared with single culture, suggesting that the interplay between EOS and HDF was essential for exacerbation of an inflammation cascade.

### IL-37b Reduced IL-31 and IL-33-Mediated Inflammation via Autophagy Mechanism Though AMPK-mTOR Signaling Pathway in EOS-HDF Co-culture

**FIGURE 1:**
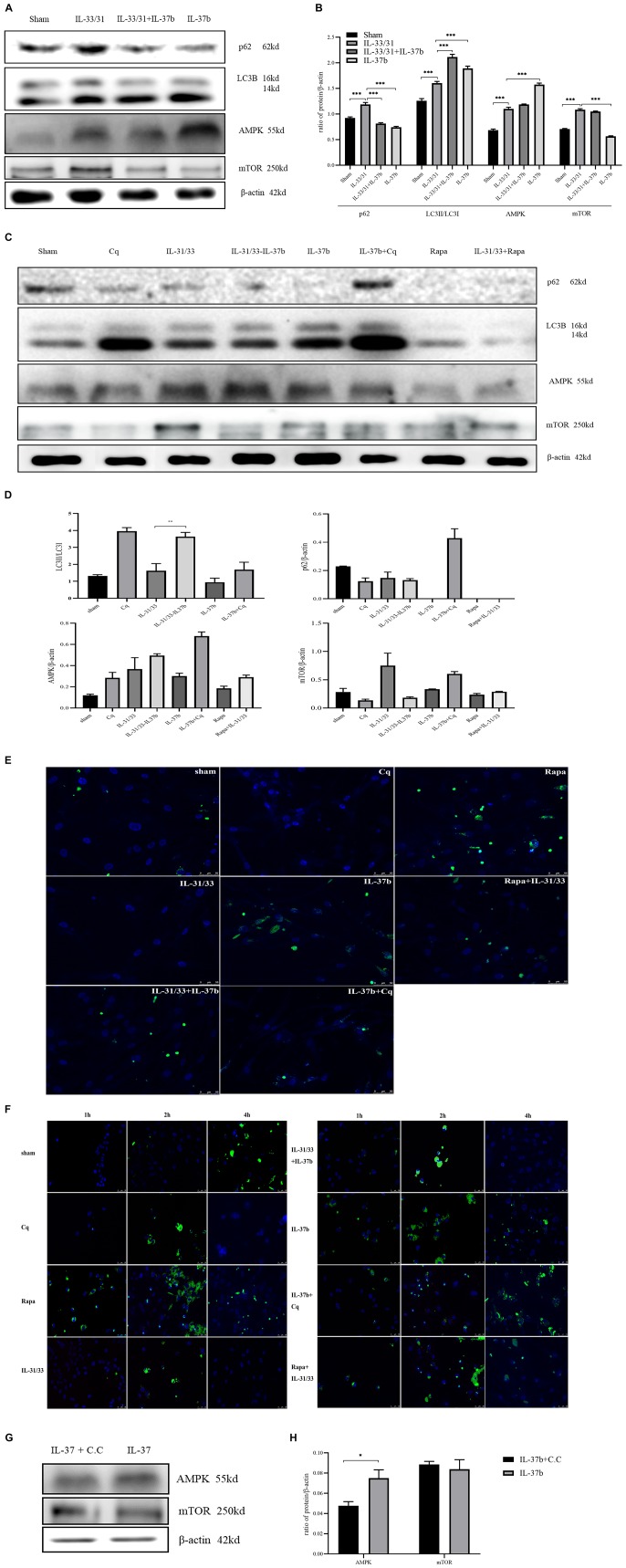
Effect of IL-37b on the induction of autophagy by analyzing the expression of p62, LC3B, AMPK and mTOR in human eosinophil-dermal fibroblast co-culture upon IL-31 (100 ng/mL) and IL-33 (100 ng/mL) stimulation. Cq: chloroquine (20 μM), autophagy negative control. Rapa: rapamycin (10 ng/mL), autophagy positive control. To verify the up-regulatory effect of IL-37b on AMPK, dorsomorphin (20 μM, Compound C/C.C), an AMPK inhibitor, together with IL-37b were applied in the co-culture. **(A)** Western blots analysis and **(B)** Quantification of AMPK, mTOR, p62 levels normalized to β-actin and LC3II/LC3I ratio (100 ng/mL IL-37b treatment for 24 h). **(C)** Western blot analysis. The Cq band for β-actin has been spliced from the same gel, due to an experimental error. Please see the Supplementary Material for the original blot image. **(D)** Quantification of AMPK, mTOR, p62 levels normalized to β-actin and LC3II/LC3I ratio in co-culture treated with IL-31, IL-33, and IL-37b (100 ng/mL), chloroquine and rapamycin treatment for 16 h. **(E)** Representative images of LC3 puncta (green) in co-culture treated with IL-31, IL-33, and IL-37b (100 ng/mL), chloroquine and rapamycin treatment for 16 h. **(F)** Representative images of LC3 puncta (green) in co-culture treated with IL-31, IL-33, and IL-37b (100 ng/mL), chloroquine and rapamycin treatment for 1, 2, and 4 h. Nuclei were visualized by DAPI (blue) staining. **(G)** Western blot analysis. **(H)** Quantification of AMPK and mTOR levels normalized to β-actin in co-culture treated with Compound C and IL-37b for 16 h. Chloroquine (Cq) pretreatment for 60 m prior to IL-37b treatment; rapamycin (Rapa) pretreatment for 10 m prior to IL-31 and IL-33 treatment. Bar charts are shown as mean ± SEM of triplicate independent experiments. *P < 0.05, **P < 0.01, and ***P < 0.001 when compared between the denoted groups.

p62, autophagy indicator of autophagy flux, can accumulate intracellularly when autophagy is inhibited and *vice versa* (17). LC3 can exist in two forms, LC3-I and LC3-II, of which LC3-II is converted from LC3-I to initiate the formation and lengthening of the autophagosome. Assessment of LC3 conversion can be used to detect autophagy. We first detected the autophagy-related protein expression in co-culture experiments (Figure 1). As shown in Figure 1A, p62 expression was increased upon IL-31 and IL-33 stimulation, while IL-37b reduced the IL-31 and IL-33-induced p62 expression. LC3-II/LC3-I expression ratio was higher in IL-37b treatment upon IL-31 and IL-33 stimulation compared with IL-31 and IL-33 stimulation alone (Figures 1A,B). Together, these results suggest that IL-37b could enhance autophagy in co-culture. Importantly, IL-37b treatment increased AMPK expression and decreased mTOR expression compared with IL-31 and IL-33 stimulation in co-culture, indicating that IL-37b could enhance autophagy through modulating AMPK and mTOR signaling pathway (18).

As shown in Supplementary Figure 4, IL-37b (50–200 ng/mL) significantly suppressed the IL-6 and CXCL8 secretion upon IL-31 and IL-33 stimulation. Moreover, mTOR inhibitor (autophagy inducer) rapamycin (rapa) treatment almost completely eliminated p62 expression and induced the conversion of LC3-I into LC3-II (Figures 1C,D), suggesting that rapamycin could increase the process of autophagy. For the treatment with IL-37b (100 ng/mL) plus downstream autophagy inhibitor chloroquine (Cq), both p62 and LC3-II accumulated while compared with IL-37b alone treatment (Figures 1C,D), demonstrating that the IL-37b-induced autophagy flux was blocked by Cq. Relatively more p62 expression and lower ratio of LC3-I/LC3-II were observed in treatment with AD-related cytokines IL-31 and IL-33, compared to IL-31 and IL-33 plus rapa treatments, suggesting that IL-31 and IL-33 could likely block autophagy. Increased AMPK and decreased mTOR expression in IL-31 and IL-33 plus IL-37b treatment were observed compared with IL-31 and IL-33 treatment, which further proved that IL-37b might enhance autophagy by upregulating AMPK and downregulating mTOR (Figures 1C,D). As shown in Figure 1E, LC3 punctas were likely not observed at 16h upon Cq treatment, while LC3 punctas were clearly observed at 16h with peak level at 2h upon rapamycin treatment (Figure 1F), demonstrating that our method and protocol were stable and reliable. In the IL-31 and IL-33 treatment group, less LC3 punctas were observed compared with the group of sham, rapamycin plus IL-31 and IL-33, or IL-31 and IL-33 plus IL-37b group, thereby suggesting that IL-31 and IL-33 could likely suppress the autophagy process. Importantly, IL-37b plus Cq treatment exhibited more LC3 punctas compared with IL-37b alone treatment. It demonstrated that autophagy flux was blocked by Cq for LC3 accumulation, and further suggested that IL-37b could enhance autophagy, which is consistent with results of the Western blot (Figures 1A–D). Together with a previous publication on its association of the autophagy-regulating inflammation (11), IL-37b could suppress inflammation by enhancing autophagy of eosinophils and dermal fibroblasts. As shown in Figures 1G,H, IL-37b plus AMPK inhibitor compound C (C.C) treatment showed less AMPK expression compared with IL-37b treatment. It has been reported that AMPK activation could suppress mTOR to enhance autophagy (19), which is consistent with our result.

### IL-37b Alleviated Circulating and *in situ* Inflammation in AD

**FIGURE 2:**
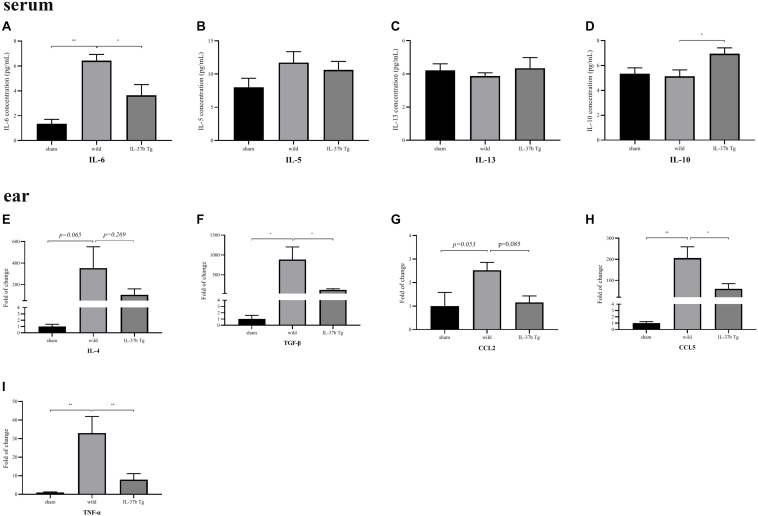
*In vivo* effect of IL-37 on the production of cytokine and chemokine in AD mice. Serum level of **(A)** IL-6, **(B)** IL-5, **(C)** IL-13, **(D)** IL-10, and mRNA expression of **(E)** IL-4, **(F)** TGF-β, **(G)** CCL2, **(H)** CCL5, and **(I)** TNF-α of wild type and IL-37b Tg mice upon MC903 stimulation. Bar charts are shown as mean ± SEM (*n* = 6 mice). *P < 0.05 and **P < 0.01 when compared between the denoted groups.

As indicated in Figure 2A, wild type AD mice showed significantly higher serum level of IL-6 compared with the sham group, suggesting severe inflammation upon MC903 stimulation. This figure also shows that IL-6 level of IL-37b transgenic (Tg) AD mice was significantly less than that of wild type AD mice, indicating that IL-37b could suppress systemic inflammation in AD mice. Meanwhile, there was no significant difference in serum levels of Th2 cytokine IL-5 and IL-13 between IL-37b Tg and wild type AD mice (Figures 2B,C, both *P* > 0.05), which is in concordance with previous study using a similar AD murine model (20). It is likely that MC903 could induce TSLP but not IL-4 derived Th2-mediated inflammation in AD (20). IL-37b Tg AD mice showed a significant upregulation of anti-inflammatory IL-10 compared with wild type AD mice (Figure 2D, *P* < 0.05). QPCR results showed that the *in situ* Th2 cytokine IL-4 mRNA expression was decreased in IL-37b Tg AD mice (Figure 2E) but significantly higher mRNA expression of TGF-β, CCL2, CCL5 and TNF-α in wild type AD mice compared with sham group, while the high level of these cytokines and chemokines was significantly reversed in IL-37b Tg AD mice (Figures 2F–I). Together, the above demonstrated that IL-37b could suppress allergic inflammation in AD.

### IL-37b Decreased Ear Swelling and Snatching Time in MC903-Induced AD

**FIGURE 3:**
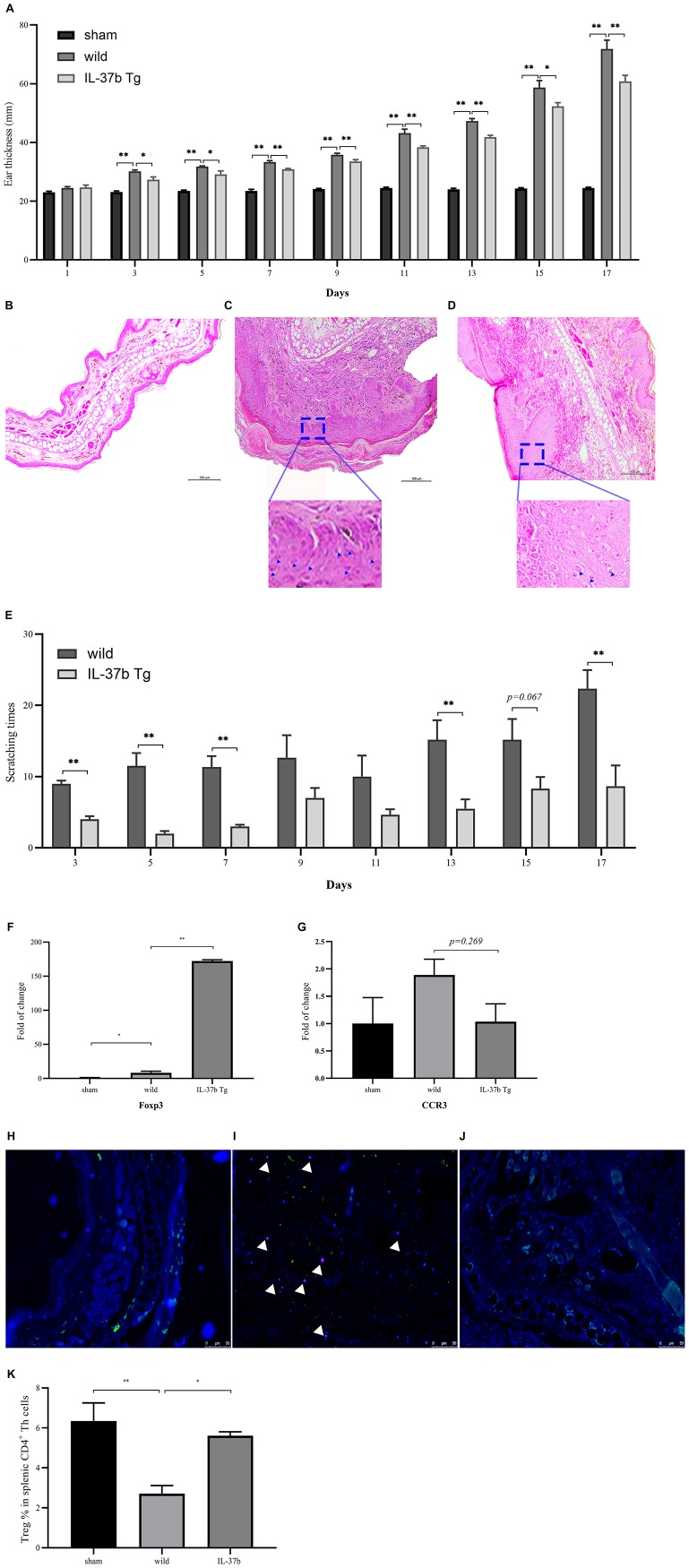
Histological assessment of AD-like skin lesions and immune cell recruitment in ear lesion tissue and spleen. **(A)** Ear swelling and H&E staining of **(B)** Sham, **(C)** Wild type, **(D)** IL-37 Tg in AD-like skin lesions, **(E)** Snatching time of wild type and IL-37b Tg mice upon MC903 stimulation. mRNA expression of **(F)** Foxp3 and **(G)** CCR3 of wild type and IL-37b Tg mice upon MC903 stimulation. Immunohistofluorescence analysis of eosinophils and dermal fibroblasts by co-staining of dermal fibroblasts and eosinophils using the anti-Vimentin (green) and anti-MBP (red; white triangle pointed) antibodies, respectively. Nuclei were visualized by DAPI (blue) staining. **(H)** Sham; **(I)** Wild type mice with MC903 stimulation; **(J)** IL-37b Tg mice with MC903 stimulation. **(K)** % of Treg cells in splenic CD4+ Th cells of different groups. Bar charts are shown as mean ± SEM (*n* = 6 mice). *P < 0.05 and **P < 0.01 when compared between the denoted groups.

As shown in Figure 3A, MC903 could induce tissue swelling and epidermal thickening. Strikingly, wild type mice receiving topical application of MC903 manifested more deteriorated AD-like symptoms, including significantly enhanced ear swelling, more extensive epidermal thickening, and the presence of higher number of eosinophils in the dermal layer (Figures 3B,C, denoted by blue triangle), and the infiltration of eosinophils into the dermal layer, compared with IL-37b Tg group (Figure 3D). IL-37 Tg mice showed significantly less ear thickness compared with wild type mice upon MC903 stimulation (Figure 3A). IL-37b also significantly ameliorated the itching state at day 3, 5, 7, 9, 13, and 17, which is an intolerable clinical feature of AD (Figure 3E).

### IL-37b Eliminated Eosinophil Infiltration in Ear Tissue Lesion and Upregulated Treg Cells in Ear and Spleen

IL-37b Tg mice showed significantly higher expression of Foxp3 compared with wild type mice upon MC903 stimulation, suggesting that more Foxp3+ Treg cells had been recruited by IL-37b into the lesion of ear to regulate inflammation (Figure 3F). Moreover, IL-37b eliminated the infiltration of CCR3+ eosinophils as shown by the down-regulation of CCR3 mRNA expression (Figures 3G–J). As shown in Supplementary Figure 3, we could deduce that HDF might be the major source for the release of pro-inflammatory cytokines and chemokines. Therefore, we suggest that IL-37b could ameliorate AD by decreasing the recruitment of eosinophils, which could stimulate HDF to release pro-inflammatory cytokines and chemokines in the lesion (Figure 3G and Supplementary Figure 3). Consistently, immunofluorescence analysis further showed that more eosinophil infiltration in the dermal layer of wild type mouse ear tissue upon MC903 stimulation than that in IL-37b Tg mice. It demonstrated that IL-37b could reduce eosinophil infiltration into AD-like skin inflammation lesion (Figures 3H–J).

Wild type AD mice (Treg/CD4^+^: 169 ± 36/6165 ± 540) showed significantly less Treg cells in spleen compared with either sham mice (Treg/CD4^+^: 532 ± 115/8519 ± 1456) and IL-37b Tg (Treg/CD4^+^: 426 ± 35/7224 ± 659) AD mice (Figure 3K and Supplementary Figure 5, all *P* < 0.05). Together with upregulating Treg cytokine IL-10 (Figure 2D), IL-37 could enhance Treg cell activity and reduce eosinophil infiltration to suppress MC903-mediated inflammation.

### IL-37b Restored Gut Microbiota Diversity in AD

For the bioinformatics analysis of the diversity of bacterial species, all effective reads were grouped by 97% DNA sequence similarity into OTUs (Operational Taxonomic Units). Statistical indices of alpha diversity are summarized in Supplementary Table 1. Results showed that MC903 stimulation or IL-37b Tg mice exhibited certain effect on Alpha Diversity. As shown in Supplementary Figure 6, the curve is nearly flat, suggesting that enough number of samples has been taken for analysis. The curve seemed flatter with the increase in sample size (Supplementary Figure 7), indicating that the sampling size was sufficient for the richness of bacterial species and diversity study.

**FIGURE 4:**
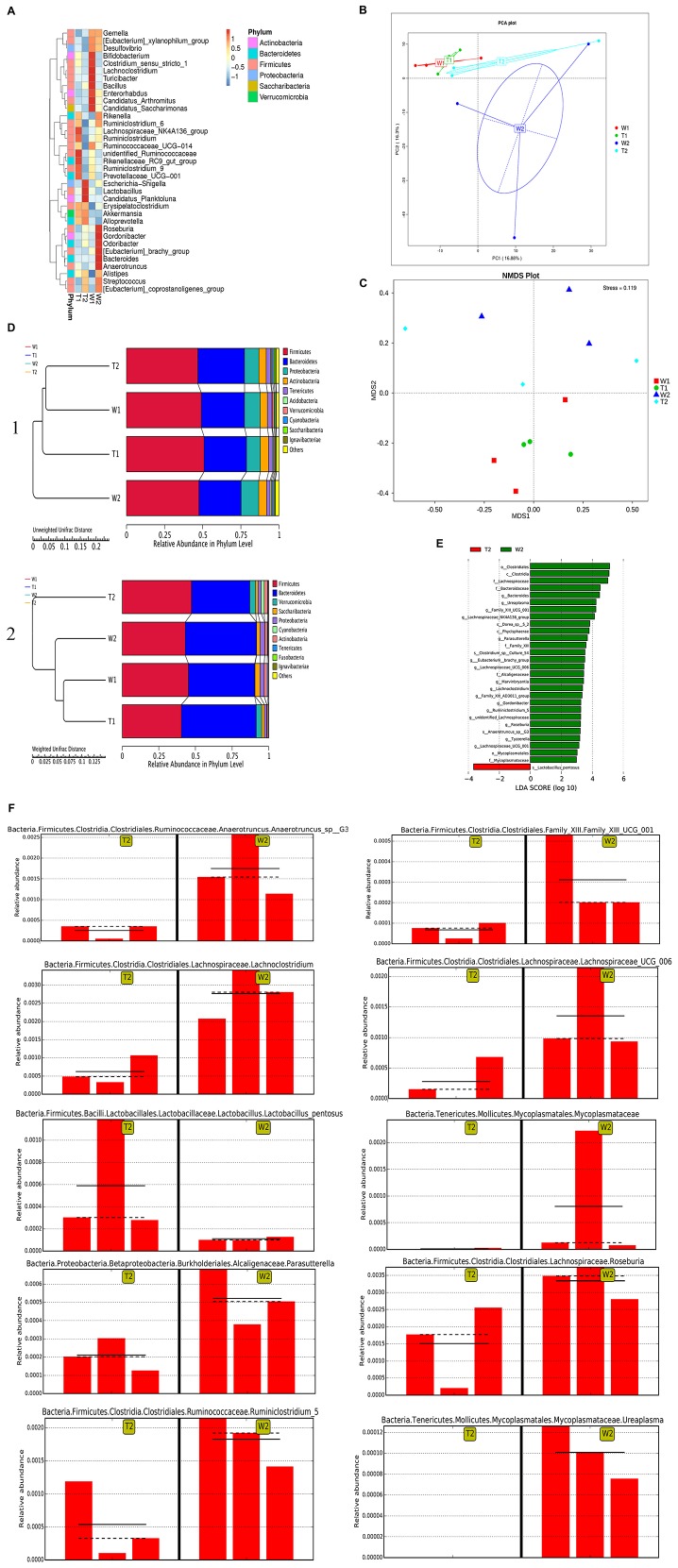
Restoration of gut microbiota by IL-37b in AD. **(A)** Species abundance heatmap of dominant 35 genera. **(B)** Principal Component Analysis (PCA). **(C)** Non-Metric Multi-Dimensional Scaling (NMDS). When the value of Stress factor is less than 0.2, it is considered that NMDS is reliable. **(D)** Unweighted Pair-group Method with Arithmetic Means (UPGMA) cluster tree based on Unweighted Unifrac distance (1) and Weighted Unifrac distance (2). **(E)** Histogram of LDA scores. **(F)** Bar chart of the relative content of representative pathogenic bacteria between W2 and T2. W1, stool samples of wild type mice at day 1 without MC903 stimulation; T1, stool samples of IL-37b Tg mice at day 1 without MC903 stimulation; W2, stool samples of wild type mice at day 17 with MC903 inducing AD model; T2, stool samples of IL-37b Tg mice at day 17 with MC903 inducing AD model. Bar charts are shown with triplicate independent experiments (*n* = 1 mouse in each experiment).

As shown in Figure 4A for the analysis of bacterial species, the abundances of roseburia, gordonbacter, odoribacter, [euvacterrium] brachy_group, bacteroides and anaerotruncus were significantly higher in W2 than that in other three groups at genus level, suggesting that they were pathological for AD. Moreover, escherichia-shigella, lactobacillus and candidatus palktoluna were significantly higher in samples of T2 group than that in other groups at genus level, suggesting IL-37 could ameliorate AD by increasing the above-mentioned bacterial species.

Clusters of W1, T1, and T2 (Figures 4B,C) samples were closely scattered. They were distinctly separated from the cluster of W2 samples, implying that MC903-induced AD had a different composition of the bacterial community and IL-37b Tg treatment had a positive therapeutic effect in the perspective of bacteria. T2 and T1 groups (Figure 4D) shared verrucomicrobia phylum, while W2 and W1 groups were absent from this phylum, thereby suggesting IL-37b might induce phylum verrucomicrobia generation for the therapeutic effect. W2 group (Figure 4D) was seen to cluster separately from other groups, this also indicated MC903-induced AD mice had an altered bacterial flora comparing with T2 groups. T2 IL-37b Tg AD group (Figure 4D) was closely clustered to W1 normal group, indicating that bacterial diversity in mice could be restored back to normal with IL-37b Tg treatment. This is consistent with the principal component analysis (PCA) and Non-Metric Multi-Dimensional Scaling (NMDA) results (Figures 4B,C).

Large number of bacterial species were screened in related paired group (Figure 4E). The detail information of the bacterial biomarkers is listed in Supplementary Table 2. Significant increases in ruminiclostridium 5, ureaplasma, lachnoclostridium, parasutterella, mycoplasmataceae, anaerotruncus, roseburia, lachnospiraceae UCG 001, lactobacillus, and Family XIII UCG 001 were observed in wild type AD mice compared with IL-37b Tg AD mice (Figure 4F). Lactobacillus pentosus significantly increased in IL-37b Tg mice compared with wild type AD mice (Figure 4F). Deferribacterales, streptococcus, opportunistic pathogens rikenella and anaeroplasma, and inflammatory bacteroides vulgatus and bifidobacteriaceae were significantly increased in wild type AD mice compared with sham (Supplementary Table 2). Wild type AD mice also showed significantly higher levels of aeromonas and arcobacter than that in IL-37b Tg AD mice. Subordinate classifications of rikenellaceae, ruminococcaceae and coriobacteriaceae were also observed with significantly high abundance in wild type AD mice (Supplementary Table 2).

### IL-37b Altered Microbiota Metabolic Pattern in AD

**FIGURE 5:**
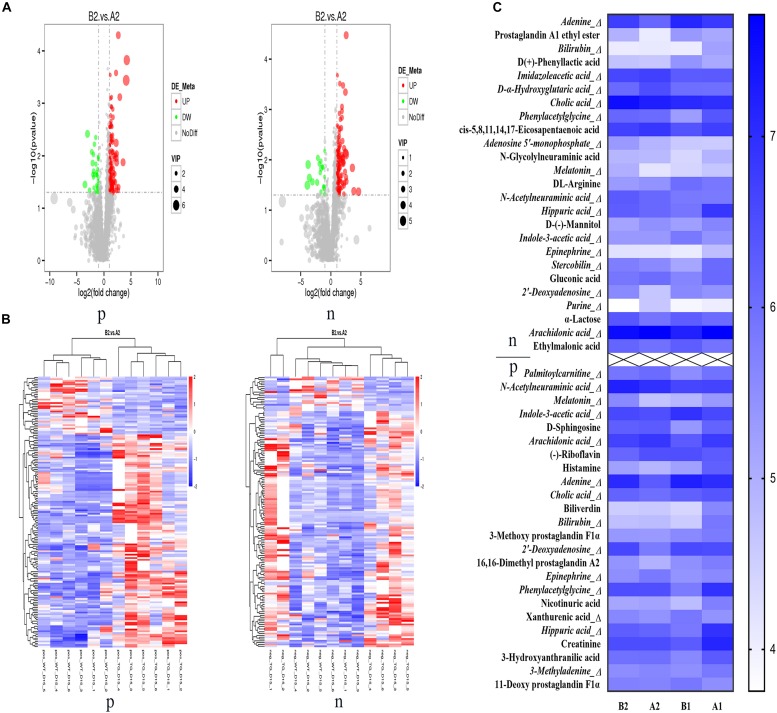
Restoration of metabolic patterns by IL-37b in AD. **(A)** Metabolite volcano map. **(B)** Metabolite clustering heat map. **(C)** Relative abundance of differential metabolites among different groups with Log_10_ of peak intensity; p: positive ion mode, n: negative ion model. A1, stool samples of wild type mice at day 1 without MC903 stimulation; B1, stool samples of IL-37b Tg mice at day 1 without MC903 stimulation; A2, stool samples of wild type mice at day 15 with MC903 inducing AD model; B2, stool samples of IL-37 Tg mice at day 15 with MC903 inducing AD model. *N* = 6 mice.

As shown by the volcano map (Figure 5A), different metabolites were screened between IL-37 Tg AD mice and wild type AD mice. From the cluster map (Figure 5B), B2, and A2 groups showed clear distinction in both ion models, suggesting that IL-37 could play a role in modulating microbial metabolites to result in a different metabolic pattern in AD. Meanwhile, A2 and A1 groups showed different metabolic patterns (Supplementary Figure 8), indicating that AD could change the microbial metabolites, which is consistent with previous clinical study (21).

The identified differential metabolites are showed in a heat map (Figure 5C) and more detailed information of metabolites is shown in Supplementary Table 3. Imidazoleacetic acid, metabolite of histamine, was significantly increased in AD mice compared with sham mice, which indicated that high levels of histamine existed in AD mice for mediating pathogenesis in AD (22). Purine could serve as a biomarker of neutrophilic airway inflammation (23), and palmitoylcarnitine could act as a potential mediator for prostate cancer progression through its effect on pro-inflammatory pathways (24). The purine and palmitoylcarnitine levels of IL-37b Tg AD mice were significantly lower than that in wild type AD mice, suggesting that IL-37 alleviated inflammation in AD was likely by suppressing purine and palmitoylcarnitine production. N-acetylneuraminic acid and xanthurenic acid were reported to prevent inflammation (25, 26). IL-37b AD mice had a higher level of N-acetylneuraminic acid and xanthurenic acid than that in wild type AD mice, implying that IL-37 might suppress inflammation by increasing N-acetylneuraminic acid and xanthurenic acid.

**FIGURE 6:**
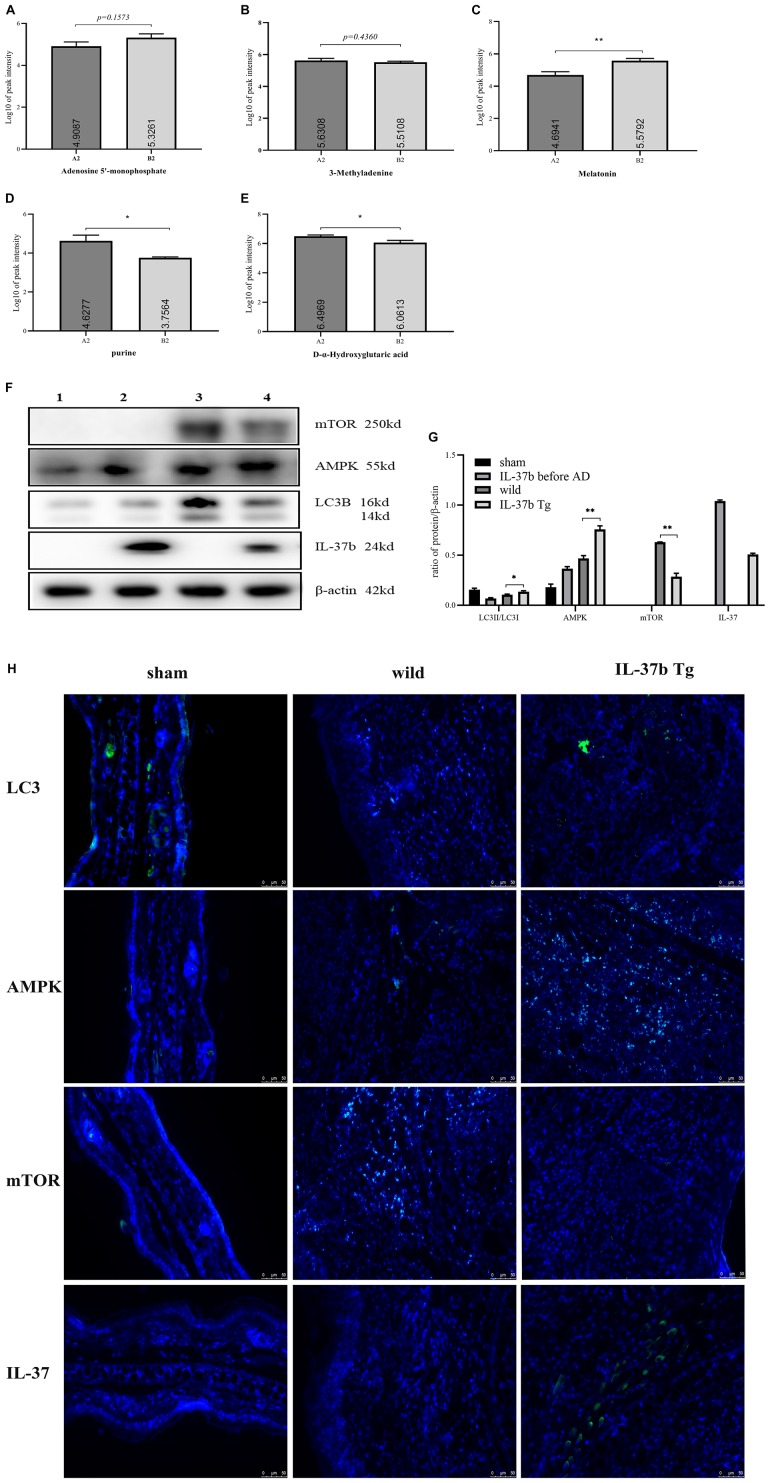
Autophagy mechanisms induced by IL-37b in AD. Autophagy related representative metabolites between different groups with Log10 of peak intensity. **(A)** Adenosine 5’-monophosphate. **(B)** 3-methyladenine. **(C)** Melatonin. **(D)** Purine. **(E)** D-α-Hydroxyglutaric acid. A2, stool samples of wild type mice at day 15 with MC903 inducing AD model; B2, stool samples of IL-37 Tg mice at day 15 with MC903 inducing AD model. **(F)** Western blot analysis of autophagy related proteins in ear tissue of wild type mice and IL-37b Tg mice, upon MC903 stimulation for 16 days and **(G)** Quantification of AMPK, mTOR, IL-37b levels normalized to β-actin and LC3II/LC3I ratio. (1: sham group, wild type mice without MC903 stimulation; 2: IL-37b non-AD group, IL-37b mice without MC903 stimulation; 3: wild type AD group, wild type mice with MC903 stimulation; 4: IL-37b Tg AD group, IL-37b Tg mice with MC903 stimulation). **(H)** Immunostaining assessment of LC3, AMPK, mTOR, and IL-37 expression in ear tissue of wild type mice and IL-37b Tg mice, upon MC903 stimulation for 16 days. Bar charts are shown as mean ± SEM (*n* = 6 mice). *P < 0.05 and **P < 0.01 when compared between the denoted groups.

IL-37b Tg AD mice showed higher cholic acid (CA) and melatonin levels than those in wild type AD mice (Figures 5C, 6C). Lower level of epinephrine in IL-37b Tg AD mice group was observed compared with wild type AD mice. Stercobilin depletion in feces was reported to represent a decrease of microbiome diversity (27). In our study, stercobilin in wild type AD mice was decreased compared with IL-37b Tg AD mice, suggesting that IL-37 could restore the normal diversity of microbiome in AD. In summary, IL-37 could suppress the inflammation in AD by regulating intestinal bacterial metabolites.

### IL-37b Enhanced Autophagy Mediated by Microbiota Metabolites Though AMPK-mTOR Signaling Pathway in AD

Increased adenosine monophosphate (AMP) was reported to induce the cell autophagy process by activating AMPK (28). IL-37b Tg AD mice had higher level of AMP than that in wild type AD mice, implying IL-37b induced autophagy (Figure 6A). 3-methyladenine (3-MA), an autophagy inhibitor, was higher in wild type AD mice than that in IL-37b Tg AD mice, suggesting that IL-37 could suppress inflammation by inducing autophagy through the downregulation of 3-MA (Figure 6B). Melatonin was reported to induce autophagy in corneal fibroblasts through mTOR-dependent pathway (29). IL-37b Tg mice showed significantly higher melatonin level than that in wild type mice to induce autophagy by increasing metabolite melatonin in AD (Figure 6C). As a natural product of purine metabolism, uric acid was reported to activate the Akt–PRAS40 pathway, which could inhibit autophagy and enhance uric acid-induced pro-inflammatory cytokines (30). As indicated in Figure 6D, IL-37b Tg mice showed significantly lower purine level compared with wild type mice. Accordingly, it is suggested that IL-37b could regulate autophagy and inflammation by suppressing purine metabolism. D-α-2-hydroxyglutarate/2-hydroxyglutarate could activate the mTOR signaling pathway to inhibit autophagy (31). The level of 2-hydroxyglutarate in IL-37b Tg mice with AD was significantly lower than that in wild type AD mice. It implicated the increased autophagy in IL-37b transgenic AD mice (Figure 6E).

In order to confirm that the autophagy was induced by IL-37 upon MC093 stimulation, we analyzed the autophagy-related proteins *in vivo*. Significantly lower ratio of LC3II/LC3I and AMPK levels, and higher mTOR level were found in the ear tissue of wild type AD mice compared with IL-37b Tg AD mice (Figures 6F,G), demonstrating that IL-37 could enhance the autophagy of the local inflammatory tissue cells through AMPK-mTOR-dependent signaling pathway in AD, which was further confirmed by immunofluorescence analysis of AMPK, mTOR, IL-37b and LC3 (Figure 6H).

## Discussion

Autophagy plays specific roles in regulating the development of immune system, promoting host innate and adaptive immune responses, and directly controlling intracellular microbes as a cellular autonomy innate defense (13). Defective autophagy can enhance the susceptibility to infection (32). Colonization of gut microbiota in the host plays a vital role in the development and maturation of the immune system, differentiation and the balance of T cells, and recruitment of immune cells upon antigen presentation, as well as the synergistic secretion of cytokines. Therefore, *in vivo* experiments with the combination of metabolomics and microbiota amplicon were conducted in this study to explore the anti-inflammatory mechanism of IL-37 for the potential treatment of AD, by focusing on the regulation of immunity through autophagy.

Autophagy could be blocked by IL-31 and IL-33 and enhanced by IL-37b in the co-culture of eosinophils and dermal fibroblasts. Autophagy is dependent on the regulation of intracellular AMPK-mTOR signaling pathway, which was confirmed through the application with inhibitor C.C (Figure 1G) and rapamycin (Figure 1C) of AMPK and mTOR, respectively. Higher ratio of LC3-II/LC3-I and decreased p62 were observed in IL-37 Tg AD mice compared with wild type AD mice (Figures 6F–G). In concordance to *in vitro* results, IL-37b Tg AD mice expressed relatively more AMPK and less mTOR compared with wild type AD mice (Figures 6F–H). Autophagy inhibitor 3-MA (Figure 6B) was decreased and the AMP (Figure 6A), the activator of AMPK for autophagy induction, was found to be significantly higher in IL-37b Tg AD mice, suggesting that IL-37 could enhance autophagy by regulating AMPK-mTOR signaling pathway. Result indicated that IL-37 could enhance the autophagy in inflammatory disease, which is consistent with the results of ear tissue protein expression.

Relationship between immunity and gut microbiota was also investigated in this study. It was elusive to interpret the anti-allergic inflammation mechanism of IL-37b regulating immunity through intestinal metabolites and bacteria in AD. Pathogens (Figure 4F and Supplementary Table 2) were screened in our study. Ruminiclostridium (33), ureaplasma (34), lachnoclostridium (35), parasutterella (36), mycoplasmataceae (37), deferribacterales (38), streptococcus (39), and aeromonas (40) were reported to act as the pathogenic source in inflammatory disease. Higher abundance of anaerotruncus, roseburia, lachnospiraceae UCG 001, lactobacillus and Family XIII UCG 001 has been observed in diabeticand obese animal models (41). Abundance of bifidobacteriaceae and diversity of intestinal microbiota were significantly lower in diabetic patients than that in healthy subjects (42). Rikenellaceae, ruminococcaceae, coriobacteriaceae and deferribacteraceae were observed with high abundance in mice on a high fat diet (43). Lactobacillus pentosus GMNL-77 was reported to inhibit skin lesions in imiquimod-induced psoriasis-like mice (44). Arcobacter species were reported to adhere to and invade eukaryotic cells, inducing an immune response and secreting toxins that damage host cells (45). Thus, by regulating the above specific bacterial species, IL-37 could reduce the inflammation and hence restore the AD mice to normal.

Gut microbiota is actually essential for the proliferation and differentiation of intra-intestinal and systemic immune effector cells. Treg cells have been shown to be decreased significantly in antibiotic-treated or germ-free animals (15). Although the detailed molecular and cellular mechanisms by which the gut microbiome influences the Treg cells are not well understood, metabolites secreted by microbiota, e.g., short chain fatty acids such as butyrate, acetate and propionate, have been shown to control the differentiation and function of mucosal Treg cells (15). In this study, we found that AD could change the microbial metabolites and IL-37 could suppress the inflammation in AD by regulating intestinal bacterial metabolites, probably via the increased the Treg cells. Metabolism of bile is affected by the lactobacillus and bifidobacteria, which can activate the relevant anti-inflammatory signaling pathway through related enzymes. In our study, primary bile acid cholic acid (CA) and lactobacillus significantly increased in IL-37b Tg AD mice compared with wild type AD mice, and the bifidobacteria abundance significantly increased in the sham group compared with AD mice (Supplementary Table 2). Catalyzed by microbial bile salt hydrolases expressed predominantly by lactobacillus and bifidobacterial, CA could be converted to the secondary bile acids, acting as NLRP3 inflammasome inhibitor, to control inflammation (46, 47). Higher CA level was also reported to positively associate with the increased Treg cytokine IL-10, enhance the generation of Foxp3+ Treg cells, reduce the AD-related CCL2 expression by recruiting fewer innate immune cells from the blood to intestine, and down-regulate the pro-inflammatory cytokine IL-6, IFN-γ, TNF-α, and IL-1β (48). Actually, anti-inflammatory serum IL-10 concentration was higher in IL-37b Tg AD mice than that in wild type AD mice (Figure 2D). Moreover, IL-37b Tg mice with or without AD showed a significantly lower expression of CCL2 (Figure 2G) and higher expression of Foxp3 (Figure 3F). Similar to our previous published results (49), the present findings further confirmed IL-37b could suppress innate immunity via the reduction of the infiltration of eosinophils and induction of Foxp3+ Treg cells, probably by regulating intestinal metabolites.

Melatonin could reduce pro-inflammatory cytokine secretion by inhibiting the adhesion of leukocytes onto endothelial cells (48), lactose (Supplementary Table 3) could promote IL-10 and suppress TNF-α and CCL2 production to reduce the infiltration of neutrophils and macrophages (50), and epinephrine could enhance the innate immune response by stimulating cytokine production from macrophages, together with upregulating CD11b expression on neutrophils (51). The above results suggest that IL-37 could indirectly modulate innate immune by the regulation of intestinal metabolites.

Combination of pruritogenic cytokine IL-31 and alarmin IL-33 were investigated in the present study because we have previously shown that they are crucial in the pathogenesis of AD (52). Keratinocyte-derived cytokine thymic stromal lymphopoietin (TSLP) is a critical mediator in the development of AD by activating dendritic cells, which in turn prime naïve T cell differentiation into Th2 cells (53). Therefore, calcemic vitamin D3 analog MC903, which can induce TSLP expression in epidermal keratinocytes and enhance Th2 inflammation in skin, was used to establish AD like murine model to evaluate the therapeutic effect of IL-37 on AD.

Remission of ear swelling was observed in IL-37b Tg groups (Figure 3A), which is in concordance with the result of reduced epidermal thickness observed in H&E staining of ear tissue presented with significantly reduced scratching frequency (Figures 3B–E). Eosinophilic infiltration in the dermal layer of ear tissue was also decreased in IL-37b Tg mice (Figures 3G–I). The remarkably down-regulated expression of Th2 cytokine IL-4 and eosinophil chemotactic protein CCL2 in ear lesion (Figures 2E,G), as well as decreasing chemokine CCL5 (Figure 2H), might account for the blockage of the infiltration of eosinophils into the dermal layer in IL-37b Tg mice upon MC903 stimulation.

For the *in vitro* and *in vivo* therapeutic potential of IL-37b on AD with its underlying mechanism of eosinophil-Th2 axis, the activation of autophagy-related AMPK-mTOR signaling pathway in eosinophil-dermal fibroblast co-culture and MC903-mediated AD like mice model, as well as the regulation of gut microbiota-immunity interaction were herein confirmed. More detailed mechanistic, clinical and translational studies are required to further confirm the application potential of IL-37 as a novel therapeutic biologic for AD.

## Data Availability Statement

The metagenomic sequencing data (16S rRNA) has been deposited in European Nucleotide Archive (ENA) with accession number PRJEB36050.

## Ethics Statement

The animal study was reviewed and approved by Animal Experimentation Ethics Committee, the Chinese University of Hong Kong, Hong Kong.

## Author Contributions

TH and C-KW designed the experiments. C-KW obtained the funding. TH, XS, JZ, K-LH, PJ, IM-TC, and MS-MT performed the experiments and analyzed the data. TH, C-KW, HZ, and CW-KL drafted and revised the manuscript.

## Conflict of Interest

The authors declare that the research was conducted in the absence of any commercial or financial relationships that could be construed as a potential conflict of interest.

## Footnotes

1 https://www.mzcloud.org/

2 http://www.chemspider.com/

## References

[1] Boguniewicz M Leung DY. Atopic dermatitis: a disease of altered skin barrier and immune dysregulation. *Immunol Rev.* (2011) 242:233–46. 10.1111/j.1600-065X.2011.01027.x 21682749 PMC3122139

[2] Weller PF Spencer LA. Functions of tissue-resident eosinophils. *Nat Rev Immunol.* (2017) 17:746–60. 10.1038/nri.2017.95 28891557 PMC5783317

[3] Kiehl P Falkenberg K Vogelbruch M Kapp A. Tissue eosinophilia in acute and chronic atopic dermatitis: a morphometric approach using quantitative image analysis of immunostaining. *Br J Dermatol.* (2001) 145:720–9. 10.1046/j.1365-2133.2001.04456.x 11736895

[4] Nold MF Nold-Petry CA Zepp JA Palmer BE Bufler P Dinarello CA. IL-37 is a fundamental inhibitor of innate immunity. *Nat Immunol.* (2010) 11:1014 10.1038/ni.1944 PMC3537119 20935647

[5] Fujita H Inoue Y Seto K Komitsu N Aihara M. Interleukin-37 is elevated in subjects with atopic dermatitis. *J Dermatol Sci.* (2013) 69:173–5. 10.1016/j.jdermsci.2012.11.001 23182761

[6] Shuai X Wei-Min L Tong Y-I Dong N Sheng Z-Y Yao Y-M. Expression of IL-37 contributes to the immunosuppressive property of human CD4+ CD25+ regulatory T cells. *Sci Rep.* (2015) 5:14478 10.1038/srep14478 PMC4585986 26411375

[7] Nold-Petry CA Lo CY Rudloff I Kirstin DE Suzhao L Michael PG IL-37 requires the receptors IL-18Rα and IL-1R8 (SIGIRR) to carry out its multifaceted anti-inflammatory program upon innate signal transduction. *Nat Immunol.* (2015) 16:354 10.1038/ni.3103 25729923

[8] Boraschi D Lucchesi D Hainzl S Maria L Elisabeth M Doris M IL-37: a new anti-inflammatory cytokine of the IL-1 family. *Eur Cytokine Netw.* (2011) 22:127–47. 10.1684/ecn.2011.0288 22047735

[9] Cavalli G Dinarello CA. Suppression of inflammation and acquired immunity by IL−37. *Immunol Rev.* (2018) 281:179–90. 10.1111/imr.12605 29247987

[10] Germic N Frangez Z Yousefi S Simon H-U. Regulation of the innate immune system by autophagy: Monocytes, macrophages, dendritic cells and antigen presentation. *Cell Death Differ.* (2019) 26:715–27. 10.1038/s41418-019-0297-6 30737475 PMC6460400

[11] Levine B Mizushima N Virgin HW. Autophagy in immunity and inflammation. *Nature.* (2011) 469:323–35. 10.1038/nature09782 21248839 PMC3131688

[12] Nakagawa I Amano A Mizushima N Akitsugu Y Hitomi Y Takahiro K Autophagy defends cells against invading group A Streptococcus. *Science.* (2004) 306:1037–40. 10.1126/science.1103966 15528445

[13] Deretic V Levine B. Autophagy, immunity, and microbial adaptations. *Cell Host Microbe.* (2009) 5:527–49. 10.1016/j.chom.2009.05.016 19527881 PMC2720763

[14] Cosmi L Liotta F Maggi E Romagnani S Annunziato F. Th17 and non-classic Th1 cells in chronic inflammatory disorders: two sides of the same coin. *Int Arch Allergy Immunol.* (2014) 164:171–7. 10.1159/000363502 25033972

[15] Noureldein MH Eid AA. Gut microbiota and mTOR signaling: Insight on a new pathophysiological interaction. *Microbial Pathogenesis.* (2018) 118:98–104. 10.1016/j.micpath.2018.03.021 29548696

[16] Zhu J Dong J Ji L Peiyong J Ting FL Dehua L Anti-allergic inflammatory activity of interleukin-37 is mediated by novel signaling cascades in human eosinophils. *Front Immunol.* (2018) 9:1445 10.3389/fimmu.2018.01445 PMC6023969 29988381

[17] Bjørkøy G Lamark T Pankiv S Øvervatn A Brech A Johansen T. Monitoring autophagic degradation of p62/SQSTM1. *Methods Enzymol.* (2009) 452:181–97. 10.1016/S0076-6879(08)03612-4 19200883

[18] Kim J Kundu M Viollet B Guan K-L. AMPK and mTOR regulate autophagy through direct phosphorylation of Ulk1. *Nat Cell Biol.* (2011) 13:132 10.1038/ncb2152 PMC3987946 21258367

[19] Alers S Löffler AS Wesselborg S Stork B. Role of AMPK-mTOR-Ulk1/2 in the regulation of autophagy: cross talk, shortcuts, and feedbacks. *Mol Cell Biol.* (2012) 32:2–11. 10.1128/mcb.06159-11 22025673 PMC3255710

[20] Naidoo K Jagot F van den Elsen L Christophe P Angela J Huijun L Eosinophils determine dermal thickening and water loss in an MC903 model of atopic dermatitis. *J Invest Dermatol.* (2018) 138:2606–16. 10.1016/j.jid.2018.06.168 29964034

[21] Huang Y Chen G Liu X Gao P Cui Z Xu G. Serum metabolomics study and eicosanoid analysis of childhood atopic dermatitis based on liquid chromatography–mass spectrometry. *J Proteome Res.* (2014) 13:5715–23. 10.1021/pr5007069 25316199

[22] Buddenkotte J Maurer M Steinhoff M. *Histamine in Inflammation.* Berlin: Springer (2010). p. 73–80.

[23] Esther C Alexis NE Clas ML Lazarowski ER Donaldson SH Pedrosa Ribeiro CM Extracellular purines are biomarkers of neutrophilic airway inflammation. *Eur Respir J.* (2008) 31:949–56. 10.1183/09031936.00089807 18256064 PMC2723793

[24] Bakheit AA Traka M Saha S Mithen R Melchini A. Accumulation of palmitoylcarnitine and its effect on pro−inflammatory pathways and calcium influx in prostate cancer. *Prostate.* (2016) 76:1326–37. 10.1002/pros.23222 27403764 PMC4996340

[25] Maes M Mihaylova I Ruyter MD Kubera M Bosmans E. The immune effects of TRYCATs (tryptophan catabolites along the IDO pathway): relevance for depression-and other conditions characterized by tryptophan depletion induced by inflammation. *Neuro Endocrinol Lett.* (2007) 28:826–31. 18063923

[26] Yida Z Imam MU Ismail M Ismail N Ideris A Abdullah MA. High fat diet-induced inflammation and oxidative stress are attenuated by N-acetylneuraminic acid in rats. *J Biomed Sci.* (2015) 22:96 10.1186/s12929-015-0211-6 PMC4619312 26498218

[27] Sekera ER Rudolph HL Carro SD Michael JM Glenna CLB Randall LR Depletion of stercobilin in fecal matter from a mouse model of autism spectrum disorders. *Metabolomics.* (2017) 13:132 10.1007/s11306-017-1277-9 PMC5685184 29147105

[28] Hardie DG. AMP-activated protein kinase—an energy sensor that regulates all aspects of cell function. *Genes Dev.* (2011) 25:1895–908. 10.1101/gad.17420111 21937710 PMC3185962

[29] Choi SI Kim KS Oh JY Jin JY Lee GH Kim EK. Melatonin induces autophagy via an mTOR-dependent pathway and enhances clearance of mutant-TGFBIp. *J Pineal Res.* (2013) 54:361–72. 10.1111/jpi.12039 23363291

[30] Crisan TO Cleophas MCP Novakovic B Kathrin E van de Veerdonk FL Stunnenberg HG Uric acid priming in human monocytes is driven by the AKT-PRAS40 autophagy pathway. *Proc Natl Acad Sci USA.* (2017) 114:5485–90. 10.1073/pnas.1620910114 28484006 PMC5448210

[31] Carbonneau M Gagné LM Lalonde M-E Germain M-A Motorina A Guiot M-C The oncometabolite 2-hydroxyglutarate activates the mTOR signalling pathway. *Nat Commun.* (2016) 7:12700 10.1038/ncomms12700 PMC5027283 27624942

[32] Shin D-M Jeon B-Y Lee H-M Jin HS Yuk J-M Song C-H Mycobacterium tuberculosis eis regulates autophagy, inflammation, and cell death through redox-dependent signaling. *PLoS Pathog.* (2010) 6:e1001230 10.1371/journal.ppat.1001230 PMC3002989 21187903

[33] Shang Q Sun W Shan X Jiang H Cai C Hao J Carrageenan-induced colitis is associated with decreased population of anti-inflammatory bacterium, Akkermansia muciniphila, in the gut microbiota of C57BL/6J mice. *Toxicol Lett.* (2017) 279:87–95. 10.1016/j.toxlet.2017.07.904 28778519

[34] Shimizu T Kida Y Kuwano K. Ureaplasma parvum lipoproteins, including MB antigen, activate NF-κB through TLR1, TLR2 and TLR6. *Microbiology.* (2008) 154:1318–25. 10.1099/mic.0.2007/016212-0 18451040

[35] Chun-Sai-Er Wang W-B Li H-Y Yi-Ming W Ma X-H Hong Yang J-M. VSL# 3 can prevent ulcerative colitis-associated carcinogenesis in mice. *World J Gastroenterol.* (2018) 24:4254 10.3748/wjg.v24.i37.4254 PMC6175759 30310258

[36] Chen YJ Wu H Wu SD Lu N Wang Y-T Liu H-N Parasutterella, in association with irritable bowel syndrome and intestinal chronic inflammation. *J Gastroenterol Hepatol.* (2018) 33:1844–52. 10.1111/jgh.14281 29744928

[37] Glavan TW Gaulke CA Rocha CS Walters SS Hirao LA Raffatellu M Gut immune dysfunction through impaired innate pattern recognition receptor expression and gut microbiota dysbiosis in chronic SIV infection. *Mucosal Immunol.* (2016) 9:677 10.1038/mi.2015.92 PMC4794436 26376368

[38] Schwab C Berry D Rauch I Rennisch I Ramesmayer J Hainzl E Longitudinal study of murine microbiota activity and interactions with the host during acute inflammation and recovery. *ISME J.* (2014) 8:1101 10.1038/ismej.2013.223 PMC3996699 24401855

[39] Wilson R Cohen JM Jose RJ de Vogel C Baxendale H Brown JS. Protection against Streptococcus pneumoniae lung infection after nasopharyngeal colonization requires both humoral and cellular immune responses. *Mucosal immunol.* (2015) 8:627 10.1038/mi.2014.95 PMC4351900 25354319

[40] Galindo CL Sha J Fadl AA Pillai LL Chopra AK. Host immune responses to Aeromonas virulence factors. *Curr Immunol Rev.* (2006) 2:13–26. 10.2174/157339506775471910

[41] Song X Zhong L Lyu N Fei L Boxing L Hao Y Inulin can alleviate metabolism disorders in ob/ob mice by partially restoring leptin-related pathways mediated by gut microbiota. *Genom Proteom Bioinformatics.* (2019) 17:64–75. 10.1016/j.gpb.2019.03.001 PMC6520907 31026583

[42] Gonai M Shigehisa A Kigawa I Kurasaki K Chonan O Matsuki T Galacto-oligosaccharides ameliorate dysbiotic Bifidobacteriaceae decline in Japanese patients with type 2 diabetes. *Benef Microbes.* (2017) 8:705–16. 10.3920/BM2016.0230 28884590

[43] Ali I Koh YS. High-fat-diet-modulated gut microbiota promotes intestinal carcinogenesis. *J Bacteriol Virol.* (2015) 45:394–6. 10.4167/jbv.2015.45.4.394

[44] Nonaka Y Izumo T Izumi F Maekawa T Shibata H Nakano A Antiallergic effects of Lactobacillus pentosus strain S-PT84 mediated by modulation of Th1/Th2 immunobalance and induction of IL-10 production. *Int Arch Allergy Immunol.* (2008) 145:249–57. 10.1159/000109294 17914277

[45] Ferreira S Queiroz JA Oleastro M Domingues FC. Insights in the pathogenesis and resistance of *Arcobacter*: a review. *Crit Rev Microbiol.* (2016) 42:364–83. 10.3109/1040841X.2014.954523 25806423

[46] Guo C Xie S Chi Z Zhang J Liu Y Zhang L Bile acids control inflammation and metabolic disorder through inhibition of NLRP3 inflammasome. *Immunity.* (2016) 45:944 10.1016/j.immuni.2016.10.009 27760343

[47] Fiorucci S Biagioli M Zampella A Distrutti E. Bile acids activated receptors regulate innate immunity. *Front Immunol.* (2018) 9:1853 10.3389/fimmu.2018.01853 PMC6099188 30150987

[48] Nabavi SM Nabavi SF Sureda A Xiao J Dehpour AR Shirooie S Anti-inflammatory effects of Melatonin: a mechanistic review. *Crit Rev Food Sci Nutr.* (2019):S4–16. 10.1080/10408398.2018.1487927 29902071

[49] Sun X Hou T Cheung EC Nga-Teng Iu T Tam VW-H Chu IM-T Anti-inflammatory mechanisms of the novel cytokine interleukin-38 in allergic asthma. *Cell Mol Immunol.* (2019) 10.1038/s41423-019-0300-7 [Epub ahead of print]. PMC7264207 31645649

[50] Pan LL Deng YY Wang R Chengfei W Jiahong L Wenying N Lactose induces phenotypic and functional changes of neutrophils and macrophages to alleviate acute pancreatitis in mice. *Front Immunol.* (2018) 9:751 10.3389/fimmu.2018.00751 PMC5913286 29719535

[51] Zhou J Yan J Liang H Jiang J. Epinephrine enhances the response of macrophages under LPS stimulation. *Biomed Res Int.* (2014) 2014:254686 10.1155/2014/254686 PMC4160625 25243125

[52] Wong CK Leung KM Qiu HN Chow JY Choi AO Lam CW. Activation of eosinophils interacting with dermal fibroblasts by pruritogenic cytokine IL-31 and alarmin IL-33: implications in atopic dermatitis. *PloS one.* (2012) 7:e29815 10.1371/journal.pone.0029815 PMC3260155 22272250

[53] Vu AT Baba T Chen X Le TA Kinoshita H Xie Y Staphylococcus aureus membrane and diacylated lipopeptide induce thymic stromal lymphopoietin in keratinocytes through the Toll-like receptor 2–Toll-like receptor 6 pathway. *J Allergy Clin Immunol.* (2010) 126:985–93. 10.1016/j.jaci.2010.09.002 21050945

