# Supplementary material for: IL-37 Ameliorating Allergic Inflammation in Atopic Dermatitis Through Regulating Microbiota and AMPK-mTOR Signaling Pathway-Modulated Autophagy Mechanism
Source: Front Immunol. 2020 Apr 28;11:752. doi: 10.3389/fimmu.2020.00752 (PMC7198885; doi:10.3389/fimmu.2020.00752)
Supplement: Supplementary file 1 [file Table_1.DOCX]

***Supplementary Figures & Tables***

**IL-37 ameliorating allergic atopic dermatitis by suppressing immune response through regulating microbiota and autophagy mechanism mediated by AMPK-mTOR signaling pathway**

Tianheng Hou^1^, Xiaoyu Sun^1^, Jing Zhu^1^, Kam-Lun Hon^2^, Peiyong Jiang^1^, Ida Miu-Ting Chu^1^, Miranda Sin-Man Tsang^1,3^, Christopher Wai-Kei Lam^4^, Huasong Zeng^5^, Chun-Kwok Wong^1,3^

^1^Department of Chemical Pathology, The Chinese University of Hong Kong, Hong Kong, China;

^2^Department of Paediatrics, The Chinese University of Hong Kong, Hong Kong, China;

^3^Institute of Chinese Medicine and State Key Laboratory of Research on Bioactivities and Clinical Applications of Medicinal Plants, The Chinese University of Hong Kong, Hong Kong, China;

^4^Faculty of Medicine and State Key Laboratory of Quality Research in Chinese Medicines, Macau University of Science and Technology, Macau, China;

^5^Department of Allergy, Immunology and Rheumatology, Guangzhou Women and Children’s Medical Center, Guangzhou Medical University, Guangzhou, Guangdong, China.

**A** **B**

**
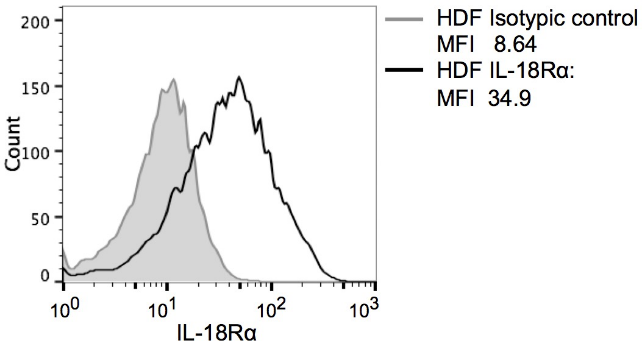

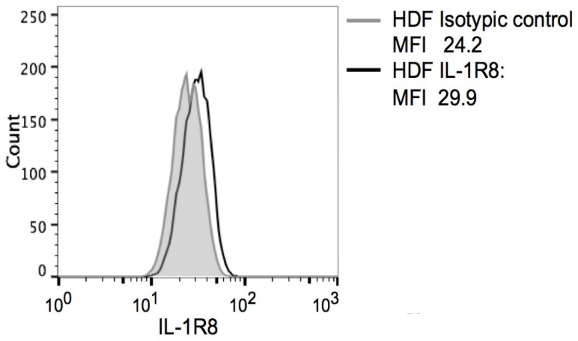
**

**Fig. 1. Expression of IL-37b receptors on HDF cells.** HDF cells were stained with (A) APC-conjugated anti-human IL-18Rα antibody and (B) PE-conjugated anti-human IL-1R8 antibody with corresponding isotypic IgG control antibody, and the expression of IL-18Rα were detected using flow cytometry (BD FACSCalibur flow cytometer) and shown in histograms.

**Fig. 2. HDF cells were cultured in 96-well plate and treated with increasing concentration of IL-37b (10 - 1000 ng/ml) or DEX (100 μg/ml) for 24 h**. After IL-37b treatment, HDF cell survival was measured by MTT colorimetric assay. Bar charts are shown as mean ± SEM. Data are representative of triplicate experiments. ****P* < 0.001 when compared between the denoted groups. DEX, dexamethasone; MTT, methyl thiazolyl tetrazolium.


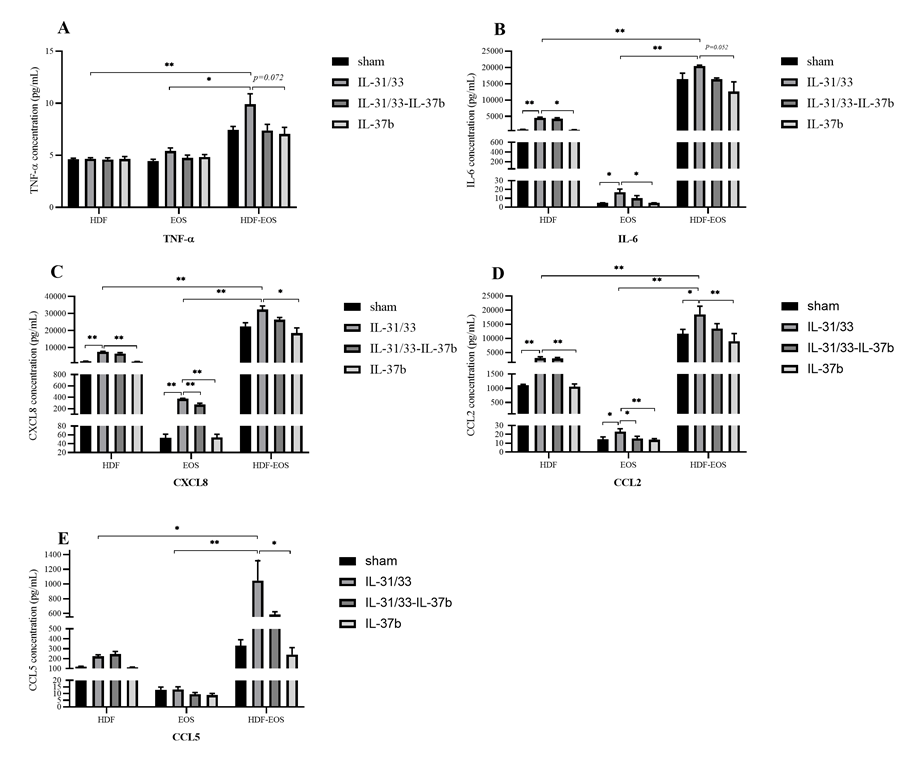


**Fig. 3. Effect of IL-37b on cytokine/chemokine release in human eosinophil-dermal fibroblast co-culture upon IL-31 and IL-33 stimulation.** Human eosinophils (3 × 10^5^) and dermal fibroblast cells (1 × 10^5^) were cultured either together or separately with or without IL-37b (100 ng/ml) pre-treatment for 10 min, followed by stimulation with IL-31 and IL-33 (100 ng/ml) for 24 h. Release of (A) TNF-α, (B) IL-6, (C) CXCL8, (D) CCL2 and (E) CCL5 in supernatant was determined. Results are shown as mean ± SEM of triplicate independent experiments. ^*^*P* < 0.05 and ^**^*P* < 0.01 when compared between the denoted groups.


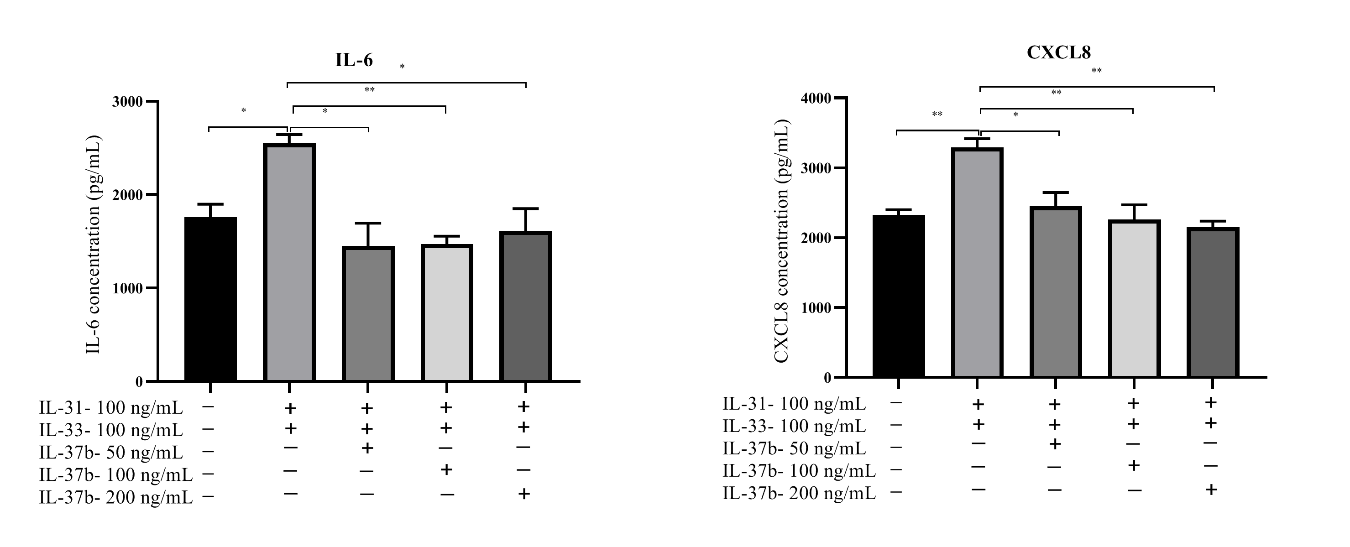


**Fig. 4.** **Effect of IL-37b on cytokine/chemokine release in human eosinophil-dermal fibroblast co-culture upon IL-31 and IL-33 stimulation.** Human eosinophils (3 × 10^5^) and dermal fibroblast cells (1 × 10^5^) were cultured either together or separately with or without IL-37b (50, 100, 200 ng/ml) pre-treatment for 10 min, followed by stimulation with IL-31 and IL-33 (100 ng/ml) for 16h. Release of IL-6 and CXCL8 in supernatant was determined. Results are shown as mean ± SEM of triplicate independent experiments. ^*^*P* < 0.05 and ^**^*P* < 0.01 when compared between the denoted groups.


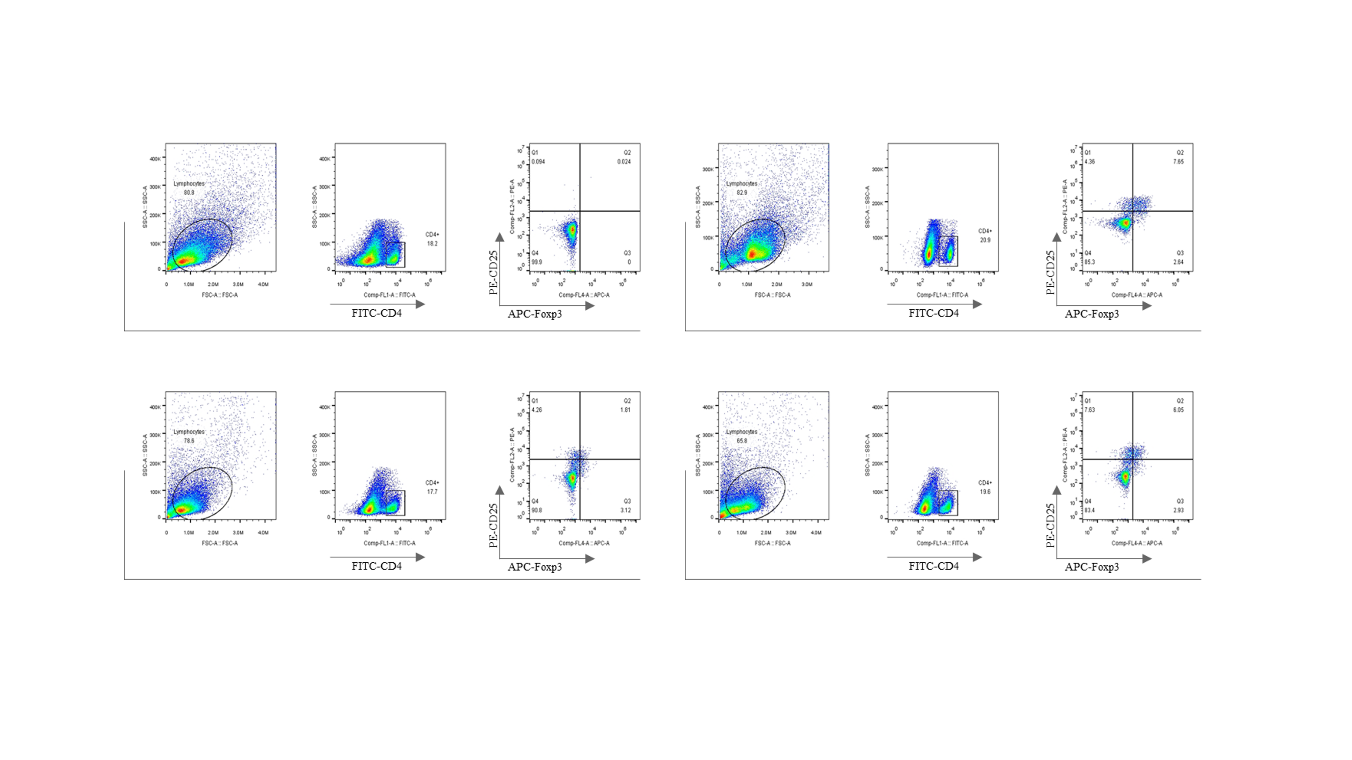


**Fig. 5. Effects of IL-37 on CD4+ Th subsets in mouse splenocyte.** Dot plots by flow cytometric analysis of Treg cell level in spleen of mice with AD. Up-left: isotype (gating strategies); up-right: Sham; down-left: wild type mice upon MC903 stimulation; down-right: IL-37b Tg mice with MC903 stimulation


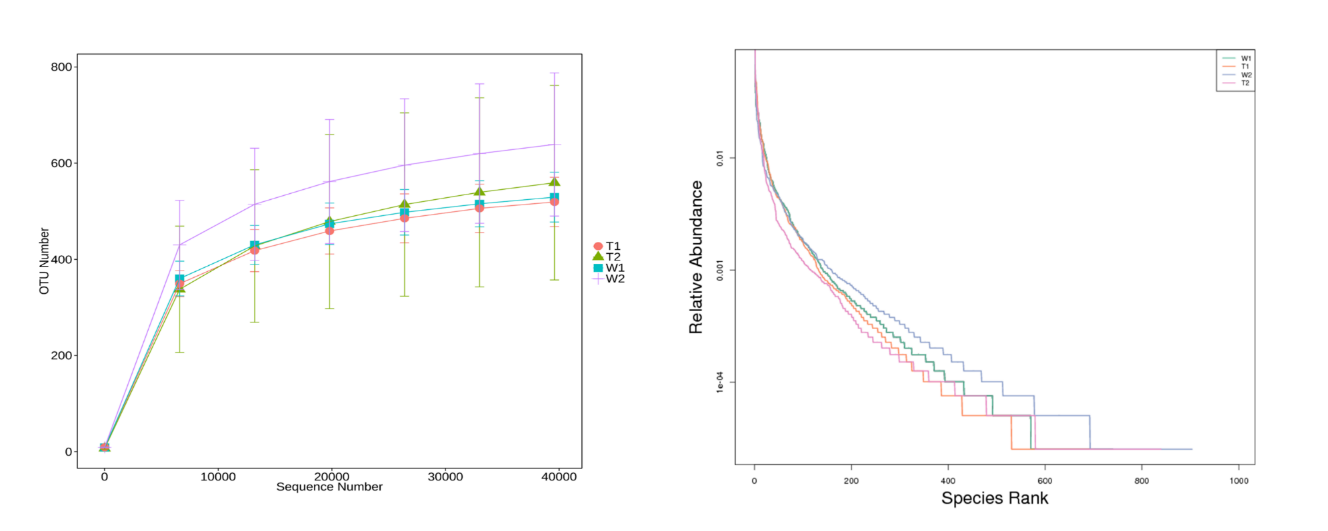
**Fig. 6. Effect of IL-37b on bacterial Alpha Diversity.** Rarefaction curves (**left**) and rank abundance curves (**right**)


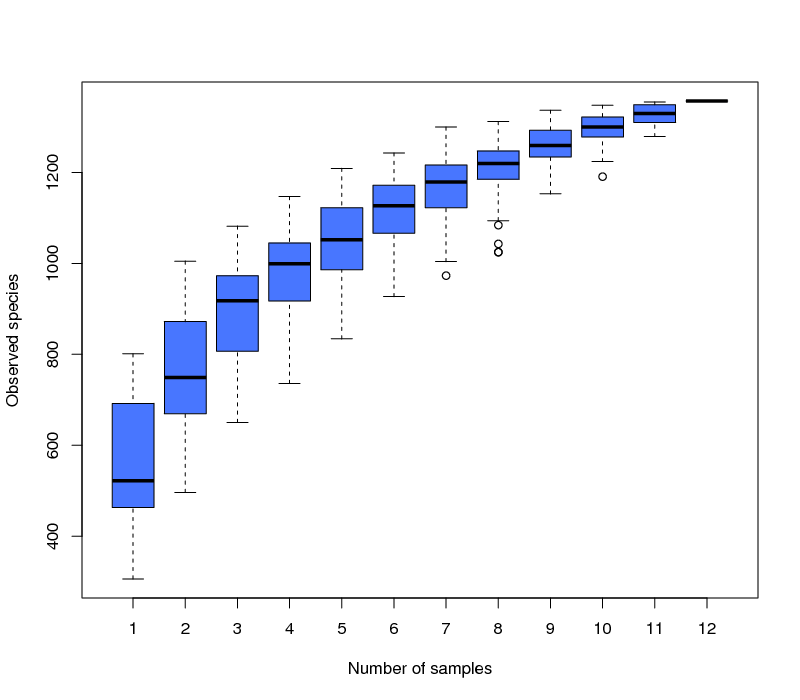


**Fig. 7. Species accumulation boxplot.** With the increase of sample size, the boxplot showing a sharp rise indicates large amount of species found in samples while the boxplot turning to flat represents adequate sample numbers.


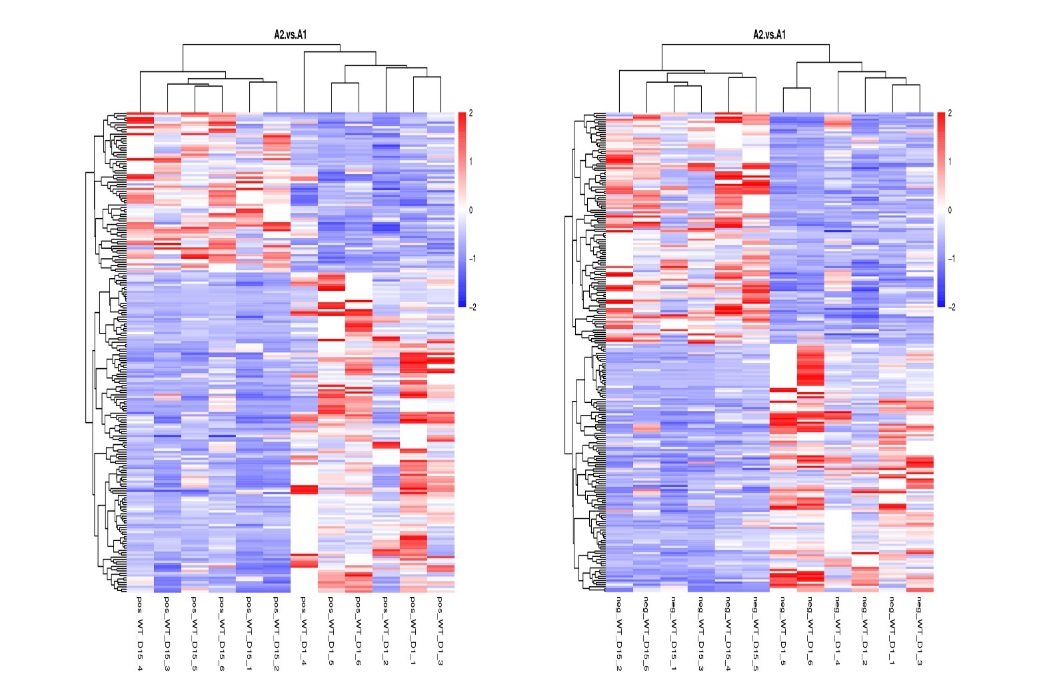


**Fig. 8. Different intestinal metabolic patterns stimulated by MC903 in AD mice.** Metabolite clustering heat map paired in A2 and A1 (**Left**: positive ion model; **right**: negative ion model). A1, stool samples of wild type mice at day 1 without MC903 stimulation; A2, stool samples of wild type mice at day 15 with MC903 inducing AD model.

**Table 1.**  Alpha Diversity Indices statistics

| **Sample** | **observed_species** | **shannon** | **simpson** | **chao1** | **ACE** | **goods_coverage** | **PD_whole_tree** |
| --- | --- | --- | --- | --- | --- | --- | --- |
| WT.D1.1 | 522 | 6.524 | 0.967 | 571.133 | 566.701 | 0.998 | 40.413 |
| WT.D1.2 | 470 | 6.193 | 0.970 | 487.466 | 497.410 | 0.999 | 51.331 |
| WT.D1.3 | 596 | 6.642 | 0.977 | 637.000 | 650.349 | 0.998 | 48.272 |
| TG.D1.1 | 508 | 6.646 | 0.979 | 563.882 | 562.053 | 0.998 | 41.375 |
| TG.D1.2 | 463 | 6.461 | 0.973 | 529.122 | 532.745 | 0.998 | 37.424 |
| TG.D1.3 | 587 | 6.163 | 0.959 | 634.879 | 629.793 | 0.998 | 51.020 |
| WT.D17.1 | 436 | 6.131 | 0.953 | 496.154 | 488.295 | 0.998 | 33.539 |
| WT.D17.2 | 692 | 6.972 | 0.977 | 757.662 | 756.246 | 0.998 | 63.921 |
| WT.D17.3 | 789 | 6.911 | 0.976 | 877.724 | 889.266 | 0.997 | 62.342 |
| TG.D17.1 | 801 | 6.616 | 0.970 | 874.598 | 878.128 | 0.997 | 71.668 |
| TG.D17.2 | 306 | 2.402 | 0.555 | 359.318 | 358.101 | 0.998 | 26.331 |
| TG.D17.3 | 571 | 6.300 | 0.969 | 680.250 | 662.506 | 0.997 | 47.093 |

**Table 2.** Different bacteria in the corresponding comparison group at different classification levels

| **Biomarker_name** | **Logarithm value** | **Groups** | **LDA_value** | **P_value** |
| --- | --- | --- | --- | --- |
| Bacteria.Bacteroidetes.Bacteroidia.Bacteroidales.Bacteroidaceae.Bacteroides | 4.9854 | W2 | 4.4678 | 0.0495 |
| Bacteria.Planctomycetes.Phycisphaerae | 1.7034 | W2 | 3.7937 | 0.0463 |
| Bacteria.Firmicutes.Clostridia.Clostridiales.Ruminococcaceae.Ruminiclostridium_5 | 3.2617 | W2 | 3.2562 | 0.0495 |
| Bacteria.Actinobacteria.Coriobacteriia.Coriobacteriales.Coriobacteriaceae.Gordonibacter | 3.6038 | W2 | 3.2597 | 0.0495 |
| Bacteria.Bacteroidetes.Bacteroidia.Bacteroidales.Bacteroidaceae | 4.9854 | W2 | 4.5058 | 0.0495 |
| Bacteria.Firmicutes.Clostridia.Clostridiales.Lachnospiraceae.Lachnospiraceae_UCG_001 | 3.3318 | W2 | 3.1404 | 0.0463 |
| Bacteria.Firmicutes.Clostridia.Clostridiales.Lachnospiraceae.Roseburia | 3.5241 | W2 | 3.2379 | 0.0495 |
| Bacteria.Firmicutes.Clostridia.Clostridiales.Family_XIII.Family_XIII_UCG_001 | 2.4935 | W2 | 4.2293 | 0.0495 |
| Bacteria.Firmicutes.Clostridia.Clostridiales.Lachnospiraceae.unidentified_Lachnospiraceae | 3.3318 | W2 | 3.2517 | 0.0495 |
| Bacteria.Firmicutes.Clostridia.Clostridiales.Ruminococcaceae.Anaerotruncus.Anaerotruncus_sp__G3 | 3.2434 | W2 | 3.2184 | 0.0495 |
| Bacteria.Firmicutes.Clostridia.Clostridiales.Lachnospiraceae | 5.3566 | W2 | 4.9988 | 0.0495 |
| Bacteria.Firmicutes.Clostridia.Clostridiales.Lachnospiraceae.Tyzzerella | 2.9076 | W2 | 3.1922 | 0.0463 |
| Bacteria.Tenericutes.Mollicutes.Mycoplasmatales.Mycoplasmataceae.Ureaplasma | 2.0045 | W2 | 4.2404 | 0.0369 |
| Bacteria.Firmicutes.Clostridia.Clostridiales.Family_XIII._Eubacterium__brachy_group | 3.8423 | W2 | 3.5143 | 0.0495 |
| Bacteria.Firmicutes.Clostridia.Clostridiales.Family_XIII | 3.9753 | W2 | 3.5918 | 0.0495 |
| Bacteria.Firmicutes.Clostridia.Clostridiales.Lachnospiraceae.Lachnoclostridium | 3.4412 | W2 | 3.3701 | 0.0495 |
| Bacteria.Proteobacteria.Betaproteobacteria.Burkholderiales.Alcaligenaceae.Parasutterella | 2.7177 | W2 | 3.6832 | 0.0495 |
| Bacteria.Firmicutes.Clostridia.Clostridiales | 5.5239 | W2 | 5.1151 | 0.0495 |
| Bacteria.Firmicutes.Clostridia.Clostridiales.Family_XIII.Family_XIII_AD3011_group | 2.6735 | W2 | 3.3378 | 0.0495 |
| Bacteria.Firmicutes.Clostridia.Clostridiales.Lachnospiraceae.Lachnospiraceae_NK4A136_group | 4.6739 | W2 | 4.1392 | 0.0495 |
| Bacteria.Proteobacteria.Betaproteobacteria.Burkholderiales.Alcaligenaceae | 2.8003 | W2 | 3.4467 | 0.0495 |
| Bacteria.Firmicutes.Clostridia.Clostridiales.Lachnospiraceae.unidentified_Lachnospiraceae.Clostridium_sp__Culture_54 | 2.6735 | W2 | 3.5213 | 0.0495 |
| Bacteria.Firmicutes.Clostridia.Clostridiales.Lachnospiraceae.Lachnospiraceae_UCG_006 | 3.1321 | W2 | 3.4590 | 0.0495 |
| Bacteria.Firmicutes.Clostridia.Clostridiales.Lachnospiraceae.Lachnoclostridium.Dorea_sp__5_2 | 3.0896 | W2 | 3.8366 | 0.0495 |
| Bacteria.Firmicutes.Clostridia | 5.5239 | W2 | 5.0795 | 0.0495 |
| Bacteria.Tenericutes.Mollicutes.Mycoplasmatales | 2.9076 | W2 | 3.0222 | 0.0463 |
| Bacteria.Tenericutes.Mollicutes.Mycoplasmatales.Mycoplasmataceae | 2.9076 | W2 | 2.9765 | 0.0463 |
| Bacteria.Firmicutes.Clostridia.Clostridiales.Lachnospiraceae.Marvinbryantia | 3.4222 | W2 | 3.4367 | 0.0495 |
| Bacteria.Firmicutes.Bacilli.Lactobacillales.Lactobacillaceae.Lactobacillus.Lactobacillus_pentosus | 2.7704 | T2 | 3.6635 | 0.0495 |

| **Biomarker_name** | **Logarithm value** | **Groups** | **LDA_value** | **P_value** |
| --- | --- | --- | --- | --- |
| Bacteria.Firmicutes.Erysipelotrichia.Erysipelotrichales.Erysipelotrichaceae.Turicibacter | 3.9076 | W1 | 3.5912 | 0.0495 |
| Bacteria.Proteobacteria.Alphaproteobacteria.Rhodobacterales.Rhodobacteraceae.Paracoccus | 1.8284 | W1 | 2.1632 | 0.0463 |
| Bacteria.Proteobacteria.Alphaproteobacteria.Rhodobacterales.Rhodobacteraceae.Paracoccus.Paracoccus_carotinifaciens | 1.8284 | W1 | 2.2167 | 0.0463 |
| Bacteria.Bacteroidetes.Sphingobacteriia.Sphingobacteriales.Chitinophagaceae | 2.7704 | W1 | 2.3302 | 0.0495 |
| Bacteria.Proteobacteria.Gammaproteobacteria.Aeromonadales.Aeromonadaceae.Aeromonas | 2.2677 | W1 | 2.3746 | 0.0463 |
| Bacteria.Firmicutes.Bacilli.Bacillales.Family_XI.Gemella | 3.6487 | W1 | 3.3388 | 0.0495 |
| Bacteria.Firmicutes.Bacilli.Lactobacillales.Streptococcaceae.Streptococcus.Streptococcus_danieliae | 2.9706 | W1 | 2.6116 | 0.0495 |
| Bacteria.Firmicutes.Clostridia.Clostridiales.Peptostreptococcaceae | 4.1388 | W1 | 3.8258 | 0.0495 |
| Bacteria.Bacteroidetes.Sphingobacteriia.Sphingobacteriales.env_OPS_17 | 2.2475 | W1 | 2.0835 | 0.0369 |
| Bacteria.Proteobacteria.Epsilonproteobacteria.Campylobacterales.Campylobacteraceae | 2.6577 | W1 | 2.4049 | 0.0495 |
| Bacteria.Firmicutes.Clostridia.Clostridiales.Clostridiaceae_1.Candidatus_Arthromitus | 4.3964 | W1 | 4.0904 | 0.0495 |
| Bacteria.Firmicutes.Bacilli.Bacillales.Family_XI | 3.6487 | W1 | 3.3408 | 0.0495 |
| Bacteria.Proteobacteria.Betaproteobacteria.Rhodocyclales | 2.5485 | W1 | 2.2530 | 0.0369 |
| Bacteria.Proteobacteria.Alphaproteobacteria.Rhodospirillales.Acetobacteraceae.Komagataeibacter | 2.2677 | W1 | 2.4209 | 0.0463 |
| Bacteria.Proteobacteria.Epsilonproteobacteria.Campylobacterales.Campylobacteraceae.Arcobacter | 2.5880 | W1 | 2.3911 | 0.0463 |
| Bacteria.Proteobacteria.Gammaproteobacteria.Pseudomonadales.Pseudomonadaceae.Pseudomonas.Pseudomonas_aeruginosa | 2.7765 | W1 | 2.4848 | 0.0463 |
| Bacteria.Bacteroidetes.Bacteroidia.Bacteroidales.Porphyromonadaceae.Odoribacter | 2.2677 | W1 | 2.0835 | 0.0369 |
| Bacteria.Firmicutes.Bacilli.Bacillales | 4.2355 | W1 | 3.8720 | 0.0495 |
| Bacteria.Proteobacteria.Betaproteobacteria.Rhodocyclales.Rhodocyclaceae | 2.5485 | W1 | 2.3064 | 0.0369 |
| Bacteria.Firmicutes.Clostridia.Clostridiales.Clostridiaceae_1.Candidatus_Arthromitus.Candidatus_Arthromitus_sp__SFB_mouse_NL | 4.3964 | W1 | 4.0880 | 0.0495 |
| Bacteria.Proteobacteria.Gammaproteobacteria.Aeromonadales | 2.3566 | W1 | 2.7565 | 0.0431 |
| Bacteria.Proteobacteria.Gammaproteobacteria.Aeromonadales.Aeromonadaceae | 2.3566 | W1 | 2.8270 | 0.0431 |
| Bacteria.Deferribacteres.unidentified_Deferribacteres.Deferribacterales.Deferribacteraceae.Mucispirillum | 1.8284 | T1 | 2.5555 | 0.0339 |
| Bacteria.Deferribacteres | 1.8284 | T1 | 2.5845 | 0.0339 |
| Bacteria.Deferribacteres.unidentified_Deferribacteres.Deferribacterales | 1.8284 | T1 | 2.4063 | 0.0339 |
| Bacteria.Bacteroidetes.Bacteroidia.Bacteroidales.Rikenellaceae.Rikenella | 3.8910 | T1 | 3.3911 | 0.0495 |
| Bacteria.Actinobacteria.Thermoleophilia.Gaiellales.Gaiellaceae | 1.9253 | T1 | 2.4550 | 0.0339 |
| Bacteria.Deferribacteres.unidentified_Deferribacteres | 1.8284 | T1 | 2.7457 | 0.0339 |
| Bacteria.Deferribacteres.unidentified_Deferribacteres.Deferribacterales.Deferribacteraceae | 1.8284 | T1 | 2.8677 | 0.0339 |
| Bacteria.Firmicutes.Clostridia.Clostridiales.Ruminococcaceae.Ruminiclostridium_1 | 2.4304 | T1 | 2.4306 | 0.0463 |
| Bacteria.Actinobacteria.Thermoleophilia.Gaiellales.Gaiellaceae.Gaiella | 1.9253 | T1 | 2.6425 | 0.0339 |
| Bacteria.Proteobacteria.Deltaproteobacteria.Desulfovibrionales.Desulfovibrionaceae.Bilophila | 2.7034 | T1 | 2.5767 | 0.0495 |
| Bacteria.Firmicutes.Clostridia.Clostridiales.Clostridiales_vadinBB60_group | 3.9864 | T1 | 3.4056 | 0.0495 |

| **Biomarker_name** | **Logarithm value** | **Groups** | **LDA_value** | **P_value** |
| --- | --- | --- | --- | --- |
| Bacteria.Firmicutes.Bacilli.Lactobacillales.Streptococcaceae | 3.7759 | W2 | 3.3574 | 0.0495 |
| Bacteria.Bacteroidetes.Bacteroidia.Bacteroidales.Bacteroidaceae.Bacteroides | 4.9854 | W2 | 4.4505 | 0.0495 |
| Bacteria.Bacteroidetes.Bacteroidia.Bacteroidales.Rikenellaceae.Rikenella.Rikenella_microfusus | 3.6101 | W2 | 3.3139 | 0.0495 |
| Bacteria.Deferribacteres | 3.0325 | W2 | 2.7862 | 0.0369 |
| Bacteria.Deferribacteres.unidentified_Deferribacteres.Deferribacterales | 3.0325 | W2 | 2.7814 | 0.0369 |
| Bacteria.Firmicutes.Erysipelotrichia.Erysipelotrichales.Erysipelotrichaceae.Faecalitalea | 3.5164 | W2 | 3.1421 | 0.0369 |
| Bacteria.Firmicutes.Bacilli.Lactobacillales.Streptococcaceae.Streptococcus | 3.7308 | W2 | 3.2987 | 0.0495 |
| Bacteria.Actinobacteria.Coriobacteriia.Coriobacteriales.Coriobacteriaceae.Gordonibacter | 3.6038 | W2 | 3.3126 | 0.0463 |
| Bacteria.Firmicutes.Clostridia.Clostridiales.Lachnospiraceae.Roseburia.Eubacterium_sp__14_2 | 3.2537 | W2 | 2.9196 | 0.0495 |
| Bacteria.Tenericutes.Mollicutes.Anaeroplasmatales | 2.9339 | W2 | 2.9975 | 0.0369 |
| Bacteria.Bacteroidetes.Bacteroidia.Bacteroidales.Rikenellaceae.Rikenella | 3.9300 | W2 | 3.4353 | 0.0495 |
| Bacteria.Bacteroidetes.Bacteroidia.Bacteroidales.Bacteroidaceae | 4.9854 | W2 | 4.4313 | 0.0495 |
| Bacteria.Firmicutes.Clostridia.Clostridiales.Ruminococcaceae._Eubacterium__coprostanoligenes_group | 3.9394 | W2 | 3.5396 | 0.0463 |
| Bacteria.Firmicutes.Clostridia.Clostridiales.Lachnospiraceae.Roseburia | 3.5241 | W2 | 3.1240 | 0.0495 |
| Bacteria.Firmicutes.Clostridia.Clostridiales.Lachnospiraceae.Lachnospiraceae_NK4A136_group.Lachnospiraceae_bacterium_A4 | 4.1195 | W2 | 3.7429 | 0.0495 |
| Bacteria.Firmicutes.Clostridia.Clostridiales.Ruminococcaceae.Anaerotruncus.Anaerotruncus_sp__G3 | 3.2434 | W2 | 2.9923 | 0.0495 |
| Bacteria.Firmicutes.Erysipelotrichia.Erysipelotrichales.Erysipelotrichaceae.Faecalitalea._Eubacterium__dolichum | 3.5164 | W2 | 3.1469 | 0.0369 |
| Bacteria.Deferribacteres.unidentified_Deferribacteres | 3.0325 | W2 | 2.8010 | 0.0369 |
| Bacteria.Tenericutes.Mollicutes.Anaeroplasmatales.Anaeroplasmataceae | 2.9339 | W2 | 2.9849 | 0.0369 |
| Bacteria.Deferribacteres.unidentified_Deferribacteres.Deferribacterales.Deferribacteraceae | 3.0325 | W2 | 2.7992 | 0.0369 |
| Bacteria.Firmicutes.Clostridia.Clostridiales.Lachnospiraceae.Blautia.Blautia_coccoides | 2.1294 | W2 | 3.4852 | 0.0495 |
| Bacteria.Bacteroidetes.Bacteroidia.Bacteroidales.Porphyromonadaceae.Odoribacter | 3.5677 | W2 | 3.2867 | 0.0495 |
| Bacteria.Firmicutes.Clostridia.Clostridiales.Ruminococcaceae.Ruminiclostridium_5 | 3.2617 | W2 | 2.8736 | 0.0495 |
| Bacteria.Firmicutes.Clostridia.Clostridiales.Christensenellaceae.Christensenellaceae_R_7_group | 2.8229 | W2 | 2.7597 | 0.0495 |
| Bacteria.Tenericutes.Mollicutes.Anaeroplasmatales.Anaeroplasmataceae.Anaeroplasma | 2.9339 | W2 | 2.9840 | 0.0369 |
| Bacteria.Proteobacteria.Deltaproteobacteria.Desulfovibrionales.Desulfovibrionaceae.Bilophila | 2.6065 | W2 | 2.5609 | 0.0495 |
| Bacteria.Actinobacteria.unidentified_Actinobacteria.Bifidobacteriales.Bifidobacteriaceae.Bifidobacterium.Bifidobacterium_thermophilum | 2.1014 | W2 | 3.3842 | 0.0369 |
| Bacteria.Firmicutes.Clostridia.Clostridiales.Lachnospiraceae.Lachnospiraceae_UCG_001 | 3.3318 | W2 | 3.0519 | 0.0495 |
| Bacteria.Bacteroidetes.Bacteroidia.Bacteroidales.Bacteroidaceae.Bacteroides.Bacteroides_vulgatus | 4.1283 | W2 | 3.8728 | 0.0495 |
| Bacteria.Deferribacteres.unidentified_Deferribacteres.Deferribacterales.Deferribacteraceae.Mucispirillum | 3.0325 | W2 | 2.7955 | 0.0369 |
| Bacteria.Bacteroidetes.Bacteroidia.Bacteroidales.Porphyromonadaceae | 3.7698 | W2 | 3.3652 | 0.0495 |
| Bacteria.Actinobacteria.unidentified_Actinobacteria.Bifidobacteriales.Bifidobacteriaceae | 3.5537 | W1 | 3.2790 | 0.0495 |
| Bacteria.Proteobacteria.Betaproteobacteria.Burkholderiales.Oxalobacteraceae | 2.5163 | W1 | 3.1948 | 0.0495 |
| Bacteria.Firmicutes.Erysipelotrichia.Erysipelotrichales.Erysipelotrichaceae.Turicibacter | 3.9076 | W1 | 3.6867 | 0.0495 |
| Bacteria.Bacteroidetes.Sphingobacteriia.Sphingobacteriales.Chitinophagaceae | 2.7704 | W1 | 2.8743 | 0.0495 |
| Bacteria.Proteobacteria.Epsilonproteobacteria.Campylobacterales.Campylobacteraceae | 2.6577 | W1 | 3.1496 | 0.0495 |
| Bacteria.Actinobacteria.unidentified_Actinobacteria.Bifidobacteriales | 3.5537 | W1 | 3.2328 | 0.0495 |
| Bacteria.Actinobacteria.unidentified_Actinobacteria.Bifidobacteriales.Bifidobacteriaceae.Bifidobacterium | 3.5537 | W1 | 3.2533 | 0.0495 |
| Bacteria.Proteobacteria.Gammaproteobacteria.Pseudomonadales.Pseudomonadaceae.Pseudomonas.Pseudomonas_aeruginosa | 2.7765 | W1 | 2.8474 | 0.0463 |
| Bacteria.Proteobacteria.Betaproteobacteria.Burkholderiales.Oxalobacteraceae.Massilia.Massilia_aurea | 2.0714 | W1 | 3.4850 | 0.0463 |
| Bacteria.Actinobacteria.unidentified_Actinobacteria.Corynebacteriales | 2.2677 | W1 | 3.3846 | 0.0495 |
| Bacteria.Proteobacteria.Alphaproteobacteria.Rickettsiales | 2.8118 | W1 | 2.8456 | 0.0495 |
| Bacteria.Proteobacteria.Epsilonproteobacteria.Campylobacterales.Campylobacteraceae.Campylobacter | 1.8284 | W1 | 3.6968 | 0.0369 |
| Bacteria.Proteobacteria.Betaproteobacteria.Burkholderiales.Oxalobacteraceae.Massilia | 2.4024 | W1 | 3.1479 | 0.0495 |

| **Biomarker_name** | **Logarithm value** | **Groups** | **LDA_value** | **P_value** |
| --- | --- | --- | --- | --- |
| Bacteria.Firmicutes.Bacilli.Lactobacillales.Streptococcaceae.Streptococcus.Streptococcus_sp__13_2043 | 2.6243 | T2 | 2.3884 | 0.0495 |
| Bacteria.Firmicutes.Erysipelotrichia.Erysipelotrichales.Erysipelotrichaceae.Faecalitalea | 2.9465 | T2 | 2.7060 | 0.0463 |
| Bacteria.Firmicutes.Clostridia.Clostridiales.Lachnospiraceae.unidentified_Lachnospiraceae.Lachnospiraceae_bacterium_A2 | 2.0045 | T2 | 2.3793 | 0.0463 |
| Bacteria.Actinobacteria.Coriobacteriia.Coriobacteriales.Coriobacteriaceae.Gordonibacter | 2.8843 | T2 | 2.6095 | 0.0463 |
| Bacteria.Tenericutes.Mollicutes.Anaeroplasmatales | 2.3055 | T2 | 2.4304 | 0.0369 |
| Bacteria.Ignavibacteriae.Ignavibacteria.Ignavibacteriales.SR_FBR_L83 | 2.3055 | T2 | 2.8422 | 0.0369 |
| Bacteria.Firmicutes.Clostridia.Clostridiales.Ruminococcaceae.Anaerotruncus.Anaerotruncus_sp__G3 | 2.4024 | T2 | 3.1681 | 0.0369 |
| Bacteria.Firmicutes.Erysipelotrichia.Erysipelotrichales.Erysipelotrichaceae.Faecalitalea._Eubacterium__dolichum | 2.9465 | T2 | 2.6735 | 0.0463 |
| Bacteria.Tenericutes.Mollicutes.Anaeroplasmatales.Anaeroplasmataceae | 2.3055 | T2 | 2.6739 | 0.0369 |
| Bacteria.Bacteroidetes.Bacteroidia.Bacteroidales.Porphyromonadaceae.Odoribacter | 3.0985 | T2 | 2.8617 | 0.0369 |
| Bacteria.Proteobacteria.Betaproteobacteria.Burkholderiales.Alcaligenaceae.Parasutterella | 2.3232 | T2 | 2.3112 | 0.0463 |
| Bacteria.Tenericutes.Mollicutes.Anaeroplasmatales.Anaeroplasmataceae.Anaeroplasma | 2.3055 | T2 | 3.0224 | 0.0369 |
| Bacteria.Firmicutes.Clostridia.Clostridiales.Lachnospiraceae.unidentified_Lachnospiraceae.Clostridium_sp_ | 2.3232 | T2 | 2.2685 | 0.0463 |
| Bacteria.Proteobacteria.Betaproteobacteria.Burkholderiales.Alcaligenaceae | 2.3877 | T2 | 2.2115 | 0.0463 |
| Bacteria.Firmicutes.Bacilli.Lactobacillales.Lactobacillaceae.Lactobacillus.Lactobacillus_faecis | 4.3505 | T2 | 4.0414 | 0.0495 |
| Bacteria.Firmicutes.Clostridia.Clostridiales.Lachnospiraceae.Lachnoclostridium.Dorea_sp__5_2 | 2.8747 | T1 | 2.3872 | 0.0495 |
| Bacteria.Firmicutes.Clostridia.Clostridiales.Ruminococcaceae | 5.0100 | T1 | 4.4580 | 0.0495 |
| Bacteria.Firmicutes.Clostridia.Clostridiales.Lachnospiraceae._Eubacterium__xylanophilum_group | 3.4888 | T1 | 3.0206 | 0.0495 |
| Bacteria.Firmicutes.Clostridia.Clostridiales.Ruminococcaceae.Ruminococcaceae_NK4A214_group | 2.5687 | T1 | 2.2269 | 0.0495 |
| Bacteria.Firmicutes.Clostridia.Clostridiales.Clostridiaceae_1 | 4.0521 | T1 | 3.5816 | 0.0495 |
| Bacteria.Firmicutes.Clostridia.Clostridiales.Lachnospiraceae._Eubacterium__ventriosum_group | 3.4082 | T1 | 3.0846 | 0.0495 |
| Bacteria.Actinobacteria.Thermoleophilia.Gaiellales.Gaiellaceae.Gaiella | 1.9253 | T1 | 2.0472 | 0.0339 |
| Bacteria.Firmicutes.Clostridia.Clostridiales.Lachnospiraceae.Coprococcus_1 | 3.4743 | T1 | 3.1021 | 0.0495 |
| Bacteria.Firmicutes.Clostridia.Clostridiales.Family_XIII.Family_XIII_UCG_001 | 2.4935 | T1 | 2.6031 | 0.0495 |
| Bacteria.Actinobacteria.Thermoleophilia.Gaiellales.Gaiellaceae | 1.9253 | T1 | 2.1452 | 0.0339 |
| Bacteria.Firmicutes.Clostridia.Clostridiales.Ruminococcaceae.Ruminococcaceae_UCG_014 | 4.6272 | T1 | 4.0883 | 0.0495 |
| Bacteria.Firmicutes.Clostridia.Clostridiales.Lachnospiraceae.Tyzzerella | 3.0683 | T1 | 2.6516 | 0.0463 |
| Bacteria.Firmicutes.Clostridia.Clostridiales.Clostridiales_vadinBB60_group.unidentified_Clostridiales_vadinBB60_group | 2.7641 | T1 | 2.4223 | 0.0495 |
| Bacteria.Firmicutes.Clostridia.Clostridiales.Lachnospiraceae.Lachnoclostridium | 3.6101 | T1 | 3.1801 | 0.0495 |
| Bacteria.Firmicutes.Clostridia.Clostridiales.Clostridiales_vadinBB60_group.unidentified_Clostridiales_vadinBB60_group.Clostridiales_bacterium_enrichment_culture_clone_06_1235251_76 | 2.7641 | T1 | 2.4373 | 0.0495 |
| Bacteria.Firmicutes.Clostridia.Clostridiales.Ruminococcaceae.Ruminiclostridium_9 | 3.9655 | T1 | 3.4993 | 0.0495 |
| Bacteria.Firmicutes.Clostridia.Clostridiales.Ruminococcaceae.Ruminiclostridium_6 | 3.7005 | T1 | 3.3170 | 0.0495 |
| Bacteria.Firmicutes.Clostridia.Clostridiales.Ruminococcaceae.Ruminiclostridium_1 | 2.4304 | T1 | 2.4093 | 0.0369 |
| Bacteria.Firmicutes.Clostridia.Clostridiales | 5.4026 | T1 | 4.8768 | 0.0495 |
| Bacteria.Proteobacteria.Betaproteobacteria.Burkholderiales.Oxalobacteraceae.Massilia.Massilia_aurea | 2.5274 | T1 | 2.2602 | 0.0495 |
| Bacteria.Firmicutes.Clostridia.Clostridiales.Clostridiales_vadinBB60_group | 3.9864 | T1 | 3.5794 | 0.0495 |
| Bacteria.Firmicutes.Clostridia.Clostridiales.Clostridiaceae_1.Clostridium_sensu_stricto_1 | 3.9864 | T1 | 3.6016 | 0.0495 |
| Bacteria.Firmicutes.Clostridia.Clostridiales.Lachnospiraceae.unidentified_Lachnospiraceae.Clostridium_sp__Culture_54 | 2.9075 | T1 | 2.5729 | 0.0495 |
| Bacteria.Firmicutes.Clostridia | 5.4027 | T1 | 4.8615 | 0.0495 |
| Bacteria.Bacteroidetes.Bacteroidia.Bacteroidales.Bacteroidaceae.Bacteroides.Bacteroides_thetaiotaomicron | 2.6577 | T1 | 2.3266 | 0.0495 |

**Table 3**. Metabolites in the corresponding comparison group

| **ID** | **Name_des** | **Formula** | **Molecular Weight** | **RT [min]** | **FC** | **log2FC** | **Pvalue** | **AUC** | **VIP** | **Up.Down** | **mzCloud score** |
| --- | --- | --- | --- | --- | --- | --- | --- | --- | --- | --- | --- |
| A2. vs. A1 | | | | | | | | | | | |
| Com_170_neg | Adenine | C5 H5 N5 | 135.05441 | 2.55 | 0.2144 | -2.2213 | 0.0070 | 0.9444 | 2.1877 | down | 93.9 |
| Com_2032_neg | Prostaglandin A1 ethyl ester | C22 H36 O4 | 364.2606 | 12.77 | 0.0643 | -3.9584 | 0.0017 | 1.0000 | 3.4401 | down | 66.3 |
| Com_2181_neg | 2'-Deoxyadenosine | C10 H13 N5 O3 | 251.10101 | 5.87 | 0.2529 | -1.9836 | 0.0158 | 0.9167 | 2.5885 | down | 71.5 |
| Com_2192_neg | Bilirubin | C33 H36 N4 O6 | 584.26274 | 12.84 | 0.2056 | -2.2820 | 0.0278 | 0.9167 | 3.3395 | down | 76.7 |
| Com_2829_neg | D(+)-Phenyllactic acid | C9 H10 O3 | 166.06254 | 1.22 | 0.3411 | -1.5519 | 0.0056 | 0.9722 | 1.4321 | down | 58.7 |
| Com_443_neg | Imidazoleacetic acid | C5 H6 N2 O2 | 126.0428 | 2.33 | 2.2104 | 1.1443 | 0.0189 | 0.8889 | 1.2163 | up | 84.7 |
| Com_597_neg | D-α-Hydroxyglutaric acid | C5 H8 O5 | 148.03694 | 1.19 | 3.4074 | 1.7687 | 0.0009 | 0.9722 | 1.8101 | up | 81.4 |
|  |  |  |  |  |  |  |  |  |  |  |  |
| B1. vs. A1 | | | | | | | | | | | |
| Com_151_neg | Hippuric acid | C9 H9 N O3 | 179.05786 | 5.53 | 0.0696 | -3.8439 | 0.0368 | 0.8333 | 2.6834 | down | 92.3 |
| Com_1658_neg | D-(-)-Mannitol | C6 H14 O6 | 182.07866 | 1.31 | 0.4503 | -1.1509 | 0.0401 | 0.8611 | 1.1189 | down | 77.5 |
| Com_2149_neg | Indole-3-acetic acid | C10 H9 N O2 | 175.06297 | 5.99 | 2.4310 | 1.2816 | 0.0257 | 0.8333 | 1.2950 | up | 59.0 |
| Com_2192_neg | Bilirubin | C33 H36 N4 O6 | 584.26274 | 12.84 | 0.0387 | -4.6933 | 0.0110 | 0.9444 | 3.0740 | down | 76.7 |
| Com_2829_neg | D(+)-Phenyllactic acid | C9 H10 O3 | 166.06254 | 1.22 | 2.2765 | 1.1868 | 0.0435 | 0.8611 | 1.1047 | up | 58.7 |
| Com_32_neg | Arachidonic acid | C20 H32 O2 | 258.2341 | 14.49 | 0.2857 | -1.8076 | 0.0135 | 0.8889 | 1.2756 | down | 83.9 |
| Com_3357_neg | N-Glycolylneuraminic acid | C11 H19 N O10 | 325.09996 | 1.23 | 0.2022 | -2.3064 | 0.0208 | 0.8889 | 1.4500 | down | 67.5 |
| Com_5362_neg | Epinephrine | C9 H13 N O3 | 183.08905 | 7.38 | 0.1317 | -2.9246 | 0.0227 | 0.8333 | 1.8435 | down | 52.2 |
| Com_673_neg | Stercobilin | C33 H46 N4 O6 | 594.34121 | 11.24 | 0.1900 | -2.3962 | 0.0266 | 0.9167 | 2.6049 | down | 73.5 |
| Com_847_neg | Gluconic acid | C6 H12 O7 | 196.05789 | 1.24 | 0.4855 | -1.0424 | 0.0297 | 0.8611 | 1.4774 | down | 54.0 |
| Com_1081_neg | Ethylmalonic acid | C5 H8 O4 | 132.04466 | 5.63 | 2.1492 | 1.1038 | 0.0399 | 0.8889 | 1.2950 | up | 54.7 |
|  |  |  |  |  |  |  |  |  |  |  |  |
| B2. vs. A2 | | | | | | | | | | | |
| Com_2032_neg | Prostaglandin A1 ethyl ester | C22 H36 O4 | 364.2606 | 12.77 | 24.4555 | 4.6121 | 0.0429 | 0.8056 | 3.7453 | up | 66.3 |
| Com_2181_neg | 2'-Deoxyadenosine | C10 H13 N5 O3 | 251.10101 | 5.87 | 5.4272 | 2.4402 | 0.0073 | 0.9444 | 4.0210 | up | 71.5 |
| Com_4628_neg | Melatonin | C13 H16 N2 O2 | 232.12046 | 8.70 | 4.1190 | 2.0423 | 0.0116 | 0.9167 | 3.4972 | up | 72.0 |
| Com_6579_neg | Purine | C5 H4 N4 | 120.04327 | 2.34 | 0.0666 | -3.9078 | 0.0320 | 0.8056 | 3.7169 | down | 52.4 |
| Com_689_neg | α-Lactose | C12 H22 O11 | 342.11544 | 1.34 | 2.4749 | 1.3074 | 0.0160 | 1.0000 | 1.9338 | up | 89.3 |
|  |  |  |  |  |  |  |  |  |  |  |  |
| B2. vs. B1 | | | | | | | | | | | |
| Com_118_neg | Cholic acid | C24 H40 O5 | 408.28679 | 11.21 | 2.2714 | 1.1836 | 0.0351 | 0.8889 | 1.5913 | up | 83.1 |
| Com_165_neg | Phenylacetylglycine | C10 H11 N O3 | 193.07355 | 6.50 | 6.5676 | 2.7154 | 0.0420 | 0.8333 | 3.0874 | up | 92.6 |
| Com_194_neg | cis-5,8,11,14,17-Eicosapentaenoic acid | C20 H30 O2 | 302.22378 | 14.23 | 2.0239 | 1.0171 | 0.0167 | 0.8889 | 1.0555 | up | 86.3 |
| Com_2151_neg | Adenosine 5'-monophosphate | C10 H14 N5 O7 P | 347.06223 | 1.44 | 3.9563 | 1.9842 | 0.0224 | 0.9167 | 2.6442 | up | 79.7 |
| Com_2829_neg | D(+)-Phenyllactic acid | C9 H10 O3 | 166.06254 | 1.22 | 0.1789 | -2.4830 | 0.0004 | 1.0000 | 2.5322 | down | 58.7 |
| Com_32_neg | Arachidonic acid | C20 H32 O2 | 258.2341 | 14.49 | 2.2195 | 1.1503 | 0.0147 | 0.9167 | 1.2037 | up | 83.9 |
| Com_3357_neg | N-Glycolylneuraminic acid | C11 H19 N O10 | 325.09996 | 1.23 | 3.3476 | 1.7431 | 0.0003 | 1.0000 | 1.8401 | up | 67.5 |
| Com_443_neg | Imidazoleacetic acid | C5 H6 N2 O2 | 126.0428 | 2.33 | 2.1568 | 1.1089 | 0.0278 | 0.8056 | 1.2171 | up | 84.7 |
| Com_4628_neg | Melatonin | C13 H16 N2 O2 | 232.12046 | 8.70 | 3.0709 | 1.6187 | 0.0330 | 0.8611 | 2.1573 | up | 72.0 |
| Com_798_neg | DL-Arginine | C6 H14 N4 O2 | 174.11127 | 1.34 | 0.1948 | -2.3601 | 0.0238 | 0.8889 | 2.5363 | down | 82.8 |
| Com_879_neg | N-Acetylneuraminic acid | C11 H19 N O9 | 309.1052 | 1.26 | 2.7676 | 1.4687 | 0.0065 | 1.0000 | 1.7930 | up | 89.3 |
|  |  |  |  |  |  |  |  |  |  |  |  |
|  |  |  |  |  |  |  |  |  |  |  |  |
| **ID** | **Name_des** | **Formula** | **Molecular Weight** | **RT [min]** | **FC** | **log2FC** | **Pvalue** | **AUC** | **VIP** | **Up.Down** | **mzCloud score** |
| A2. vs. A1 | | | | | | | | | | | |
| Com_289_pos | Histamine | C5 H9 N3 | 111.07969 | 1.04 | 0.0288 | -5.1194 | 0.0100 | 0.9444 | 4.1256 | down | 73.9 |
| Com_432_pos | Adenine | C5 H5 N5 | 135.05471 | 1.38 | 0.1961 | -2.3500 | 0.0162 | 0.9167 | 2.3095 | down | 86.9 |
| Com_5310_pos | Cholic Acid | C24 H40 O5 | 425.31346 | 12.95 | 0.4319 | -1.2111 | 0.0027 | 0.9444 | 1.1621 | down | 82.4 |
| Com_5322_pos | Biliverdin | C33 H34 N4 O6 | 582.24756 | 15.70 | 0.1540 | -2.6993 | 0.0460 | 0.8333 | 3.3251 | down | 87.6 |
| Com_5323_pos | Bilirubin | C33 H36 N4 O6 | 584.26323 | 15.70 | 0.1475 | -2.7615 | 0.0365 | 0.8611 | 3.4467 | down | 91.4 |
| Com_554_pos | 3-Methoxy prostaglandin F1α | C21 H38 O6 | 336.22945 | 13.58 | 0.3087 | -1.6956 | 0.0460 | 0.8056 | 1.5107 | down | 52.7 |
| Com_572_pos | 2'-Deoxyadenosine | C10 H13 N5 O3 | 251.10173 | 1.65 | 0.3212 | -1.6385 | 0.0479 | 0.8611 | 2.2747 | down | 92.2 |
| Com_617_pos | 16,16-Dimethyl prostaglandin A2 | C22 H34 O4 | 362.24516 | 12.77 | 0.0799 | -3.6463 | 0.0179 | 1.0000 | 2.5092 | down | 67.2 |
|  |  |  |  |  |  |  |  |  |  |  |  |
| B1. vs. A1 | | | | | | | | | | | |
| Com_130_pos | Phenylacetylglycine | C10 H11 N O3 | 193.07408 | 8.03 | 0.0542 | -4.2060 | 0.0180 | 0.8889 | 3.7313 | down | 90.3 |
| Com_131_pos | Palmitoylcarnitine | C23 H45 N O4 | 399.33448 | 13.24 | 0.3693 | -1.4370 | 0.0018 | 0.9722 | 1.3505 | down | 95.4 |
| Com_148_pos | Nicotinuric acid | C8 H8 N2 O3 | 180.05363 | 6.70 | 0.1012 | -3.3053 | 0.0301 | 0.8889 | 3.1968 | down | 57.3 |
| Com_26_pos | Xanthurenic acid | C10 H7 N O4 | 205.03759 | 8.64 | 2.8341 | 1.5029 | 0.0110 | 0.9167 | 1.6897 | up | 84.3 |
| Com_289_pos | Histamine | C5 H9 N3 | 111.07969 | 1.04 | 0.0614 | -4.0259 | 0.0345 | 0.8611 | 3.1088 | down | 73.9 |
| Com_291_pos | Hippuric acid | C9 H9 N O3 | 179.05838 | 7.39 | 0.0628 | -3.9937 | 0.0289 | 0.8333 | 3.6282 | down | 83.4 |
| Com_327_pos | D-Sphingosine | C18 H37 N O2 | 299.28208 | 12.84 | 0.0867 | -3.5281 | 0.0224 | 0.8889 | 3.2458 | down | 73.4 |
| Com_362_pos | Creatinine | C4 H7 N3 O | 113.05906 | 1.23 | 0.1223 | -3.0314 | 0.0330 | 0.8611 | 2.8531 | down | 90.9 |
| Com_407_pos | Arachidonic acid | C20 H32 O2 | 304.23991 | 15.07 | 0.2442 | -2.0342 | 0.0492 | 0.8333 | 1.7570 | down | 90.5 |
| Com_5322_pos | Biliverdin | C33 H34 N4 O6 | 582.24756 | 15.70 | 0.0286 | -5.1259 | 0.0152 | 0.9167 | 3.8625 | down | 87.6 |
| Com_5323_pos | Bilirubin | C33 H36 N4 O6 | 584.26323 | 15.70 | 0.0256 | -5.2863 | 0.0086 | 0.9167 | 4.1317 | down | 91.4 |
| Com_559_pos | 3-Hydroxyanthranilic acid | C7 H7 N O3 | 153.04276 | 1.89 | 0.1143 | -3.1288 | 0.0329 | 0.8611 | 2.3442 | down | 84.5 |
| Com_593_pos | 3-Methyladenine | C6 H7 N5 | 149.07034 | 1.37 | 0.3793 | -1.3986 | 0.0455 | 0.8056 | 1.0981 | down | 68.4 |
| Com_642_pos | 11-Deoxy prostaglandin F1α | C20 H36 O4 | 340.26098 | 14.49 | 2.1320 | 1.0922 | 0.0136 | 0.9167 | 1.1273 | up | 62.5 |
|  |  |  |  |  |  |  |  |  |  |  |  |
| B2. vs. A2 | | | | | | | | | | | |
| Com_184_pos | Melatonin | C13 H16 N2 O2 | 232.12111 | 8.71 | 4.8202 | 2.2691 | 0.0057 | 0.9444 | 4.1653 | up | 64.6 |
| Com_322_pos | Epinephrine | C9 H13 N O3 | 183.08972 | 5.90 | 0.4677 | -1.0963 | 0.0378 | 0.8056 | 1.5650 | down | 54.0 |
| Com_432_pos | Adenine | C5 H5 N5 | 135.05471 | 1.38 | 4.9662 | 2.3121 | 0.0111 | 0.9722 | 3.5890 | up | 86.9 |
| Com_572_pos | 2'-Deoxyadenosine | C10 H13 N5 O3 | 251.10173 | 1.65 | 3.3926 | 1.7624 | 0.0302 | 0.8056 | 3.7635 | up | 92.2 |
| Com_617_pos | 16,16-Dimethyl prostaglandin A2 | C22 H34 O4 | 362.24516 | 12.77 | 3.7759 | 1.9168 | 0.0456 | 0.8056 | 2.0945 | up | 67.2 |
|  |  |  |  |  |  |  |  |  |  |  |  |
| B2. vs. B1 | | | | | | | | | | | |
| Com_131_pos | Palmitoylcarnitine | C23 H45 N O4 | 399.33448 | 13.24 | 2.3255 | 1.2175 | 0.0011 | 1.0000 | 1.6849 | up | 95.4 |
| Com_162_pos | N-Acetylneuraminic acid | C11 H19 N O9 | 309.10577 | 1.44 | 3.8141 | 1.9313 | 0.0001 | 1.0000 | 2.7423 | up | 94.8 |
| Com_184_pos | Melatonin | C13 H16 N2 O2 | 232.12111 | 8.71 | 3.4886 | 1.8026 | 0.0157 | 0.8889 | 3.1718 | up | 64.6 |
| Com_271_pos | Indole-3-acetic acid | C10 H9 N O2 | 175.06349 | 9.51 | 0.4854 | -1.0428 | 0.0151 | 0.9167 | 1.5934 | down | 87.4 |
| Com_327_pos | D-Sphingosine | C18 H37 N O2 | 299.28208 | 12.84 | 6.3568 | 2.6683 | 0.0008 | 0.9722 | 3.9416 | up | 73.4 |
| Com_407_pos | Arachidonic acid | C20 H32 O2 | 304.23991 | 15.07 | 2.4256 | 1.2783 | 0.0165 | 0.8889 | 1.7783 | up | 90.5 |
| Com_5548_pos | (-)-Riboflavin | C17 H20 N4 O6 | 376.13797 | 8.08 | 0.3359 | -1.5739 | 0.0438 | 0.9167 | 1.6639 | down | 87.4 |

Note：B2 vs. B1 up: B2> B1; B2 vs. B1 down: B2< B1
