# Supplementary material for: IL-37 Ameliorating Allergic Inflammation in Atopic Dermatitis Through Regulating Microbiota and AMPK-mTOR Signaling Pathway-Modulated Autophagy Mechanism
Source: Front Immunol. 2020 Apr 28;11:752. doi: 10.3389/fimmu.2020.00752 (PMC7198885; doi:10.3389/fimmu.2020.00752)
Supplement: Supplementary file 2 [file Data_Sheet_1.DOCX]

**Raw Western blot of Figure 1E**

**IL-37 ameliorating allergic inflammation in atopic dermatitis through regulating microbiota and AMPK-mTOR signaling pathway-modulated autophagy mechanism**

Tianheng Hou^1^, Xiaoyu Sun^1^, Jing Zhu^1^, Kam-Lun Hon^2^, Peiyong Jiang^1^, Ida Miu-Ting Chu^1^, Miranda Sin-Man Tsang^1,3^, Christopher Wai-Kei Lam^4^, Huasong Zeng^5^, Chun-Kwok Wong^1,3^


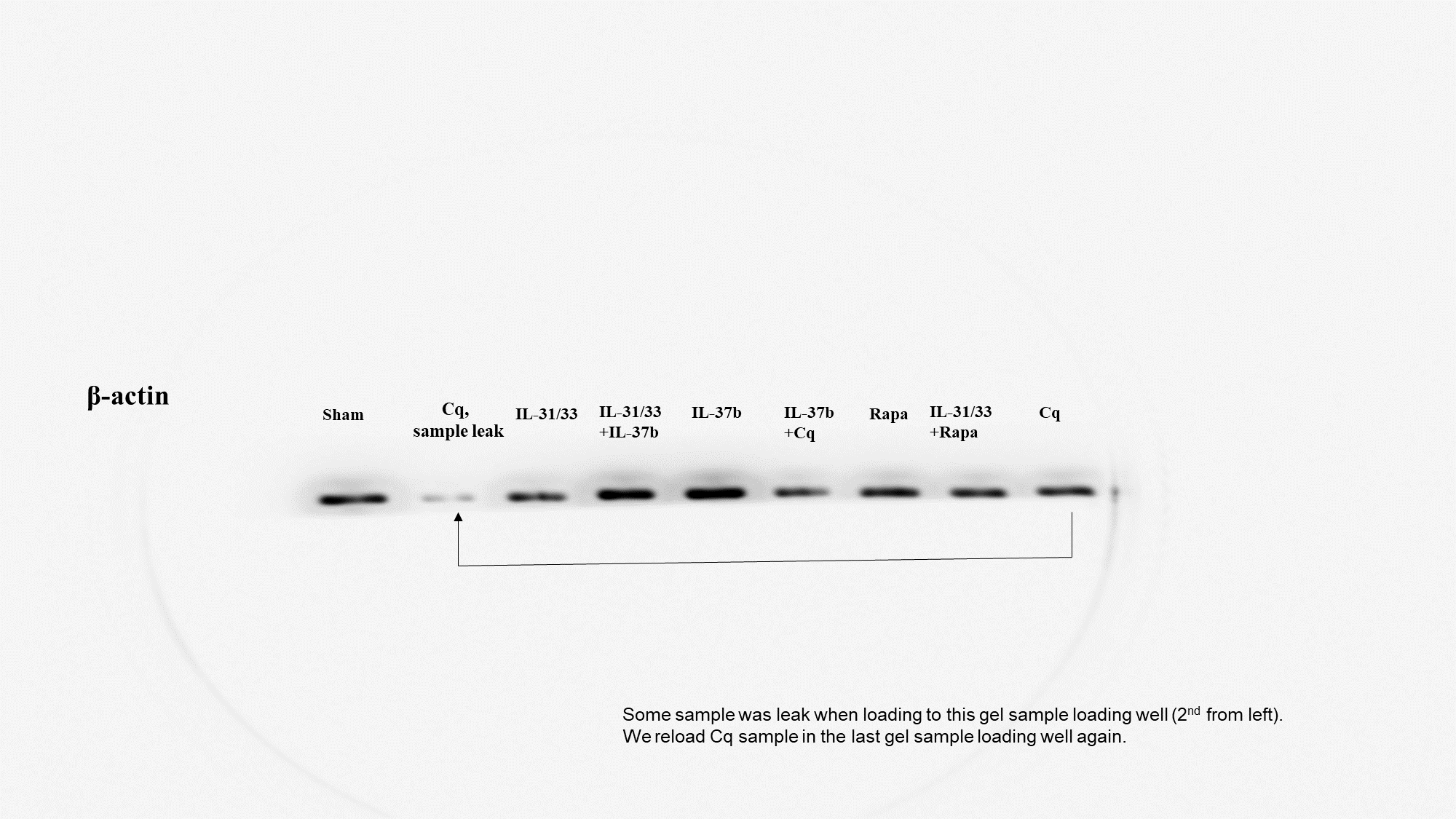
Figure 1E (β-actin)

Some protein sample (spilled out) leaked when loaded to this gel sample loading well (2^nd^ from left). Therefore, we reloaded Cq treated sample in the right end sample loading well again.
